# In vivo production of RNA nanostructures via programmed folding of single-stranded RNAs

**DOI:** 10.1038/s41467-018-04652-4

**Published:** 2018-06-06

**Authors:** Mo Li, Mengxi Zheng, Siyu Wu, Cheng Tian, Di Liu, Yossi Weizmann, Wen Jiang, Guansong Wang, Chengde Mao

**Affiliations:** 1 Department of Chemistry, Purdue University, West Lafayette, IN 47907 USA; 2 Department of Chemistry, University of Chicago, Chicago, IL 60637 USA; 3 Markey Center for Structural Biology and Department of Biological Sciences, Purdue University, West Lafayette, IN 47907 USA; 4 The Institute of Respiratory Diseases, Xinqiao Hospital, 400037 Chongqing, China

## Abstract

Programmed self-assembly of nucleic acids is a powerful approach for nano-constructions. The assembled nanostructures have been explored for various applications. However, nucleic acid assembly often requires chemical or in vitro enzymatical synthesis of DNA or RNA, which is not a cost-effective production method on a large scale. In addition, the difficulty of cellular delivery limits the in vivo applications. Herein we report a strategy that mimics protein production. Gene-encoded DNA duplexes are transcribed into single-stranded RNAs, which self-fold into well-defined RNA nanostructures in the same way as polypeptide chains fold into proteins. The resulting nanostructure contains only one component RNA molecule. This approach allows both in vitro and in vivo production of RNA nanostructures. In vivo synthesized RNA strands can fold into designed nanostructures inside cells. This work not only suggests a way to synthesize RNA nanostructures on a large scale and at a low cost but also facilitates the in vivo applications.

## Abstract (web summary)

RNA nanostructures have been demonstrated in a range of biological applications, but their assembly and delivery to cells is difficult. Here the authors demonstrate the in vivo assembly of a RNA nanostructure from a single transcript inside the cellular environment.

## Introduction

Nucleic acids (DNA and RNA) have been extensively explored for molecular self-assembly and a wide range of nanostructures have been assembled from nucleic acids.^1–9^ Such nanostructures can be applied to various fields from physical devices to biomedical applications.^10–13^ Following DNA nanotechnology, programmed RNA self-assembly has rapidly evolved in hope that RNA has more structural complexity and functional diversity. Up to now, DNA/RNA self-assembly generally starts from chemically or enzymatically synthesized single-stranded DNA or RNA (ssDNA or ssRNA). This method is not desirable for large-scale production because of the excessive cost. A potential solution is to clone nucleic acids in bacteria, such as *E. coli*, in the same way as recombinant proteins.^14, 15^ However, DNA molecules normally exist as duplexes in the cell. Though direct cellular production of ssDNAs is possible in some special cases such as by reverse transcriptase^16, 17^ or M13 bacterial genomes^5^, there are limitations. For instance, the ssDNA length range is limited or additional enzymatic treatment after purification is needed.^18^ Comparatively, a more suitable choice is RNA. Cellular RNAs primarily exist as single-stranded, and their length can vary in a broad range. Recently, Geary et al. have demonstrated that ssRNAs, in test-tubes, can cotranscriptionally fold into designed nanomotifs, which can further assemble into 2D arrays.^9^ Delebecque et al. have cloned RNA nanostructures in *E. coli* to organize chemical reactions in vivo.^12^ However, the nanoscaled, structural details of the RNA complexes have not been thoroughly characterized under native conditions.

In this work, we have developed a versatile strategy to prepare well-defined nanostructures by folding individual long ssRNAs. Each nanostructure contains only one ssRNA molecule. The resulting nanostructures can be cloned, expressed, and self-folded in *E. coli.* RNA nanostructures have been thoroughly characterized by gel electrophoresis, atomic force microscopy (AFM) imaging, and cryogenic electron microscopy (cryoEM).

A key challenge of this approach is to design the folding pathway to avoid kinetic traps. For nucleic acid self-assembly, the target structures are designed to be thermodynamically stable, but often not kinetically favored. This problem is commonly solved by slowly cooling the samples from a high temperature (e.g., 95 °C) to a low temperature (e.g., 25 °C) over a long period of time.^19^ Obviously, this thermal annealing process is not feasible for nucleic acid self-folding in vivo. A potential approach is to design the targeted nucleic acid nanostructures both thermodynamically stable and kinetically favorable. To achieve this goal, the ssRNA is designed to fold following a sequential and hierarchical pathway. Newly synthesized ssRNA would first fold into hairpins while transcription. Hairpin structures are not only thermodynamically stable but also topologically simple. They only involve local interactions, thus, fold quickly. If any alternative structure forms, it would readily rearrange into the target hairpin structure via local branch migration.^20^ Upon hairpin formation, which defines the RNA’s secondary structure, most of the RNA residues are inert as being in the content of duplexes, leaving a minimal amount of RNA residues as unpaired. The unpaired residues are able to further form long-range tertiary interactions, leading to the formation of fully folded, designed nanostructures. The overall folding pathway is similar to that of the naturally occurring complex RNA structures, such as hairpin ribozymes.^21^. Conceptually, the design concept resembles the principle that developed by Geary et al. However, a significant change is that the short dovetail seams (2–3 bps) are avoided. Such short helical domains are not very stable and are likely to deformation under mild stress.

## Results

### Molecular design

**Fig. 1:**
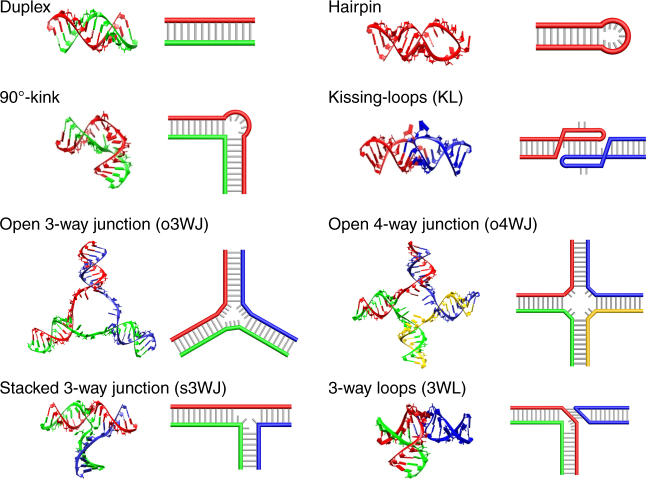
Component motifs of RNA structures. For each motif, a schematic drawing and a 3D model are shown. The thick colored lines and thin gray lines represent RNA backbones and basepairs, respectively

The RNA nanostructures in this study are rationally designed based on natural RNA motifs and tertiary interactions (Fig. 1), including: (i) RNA duplexes, (ii) RNA hairpins^22^, (iii) 3-way junctions in open conformation (o3WJ)^23^, (vi) 3-way junctions in stacked conformation (s3WJ) observed in the packaging RNA (pRNA) of phi29 bacteriophage,^24^ (v) coaxially stacked kissing loops (KLs) found in the dimerization initiation sites of HIV-1 RNA,^25^ (vi) a 3-way loop (3WL) interaction observed in phi29 pRNA,^26^ (vii) 4-way junctions in open conformation (o4WJ), and (viii) 90°–kink found in the internal ribosome entry site (IRES) of the hepatitis C virus (HCV) RNA genome.^27^ According to the molecular designs, DNA templates coding for the ssRNAs have been synthesized so that RNAs can be produced by in vitro transcription. Alternatively, the DNA templates can be inserted into a plasmid and introduced into *E. coli.* Then cellular transcription machinery will transcribe the gene into the corresponding ssRNA, which spontaneously folds into the designed nanostructures inside the cells.

### RNA square and double-square

**Fig. 2:**
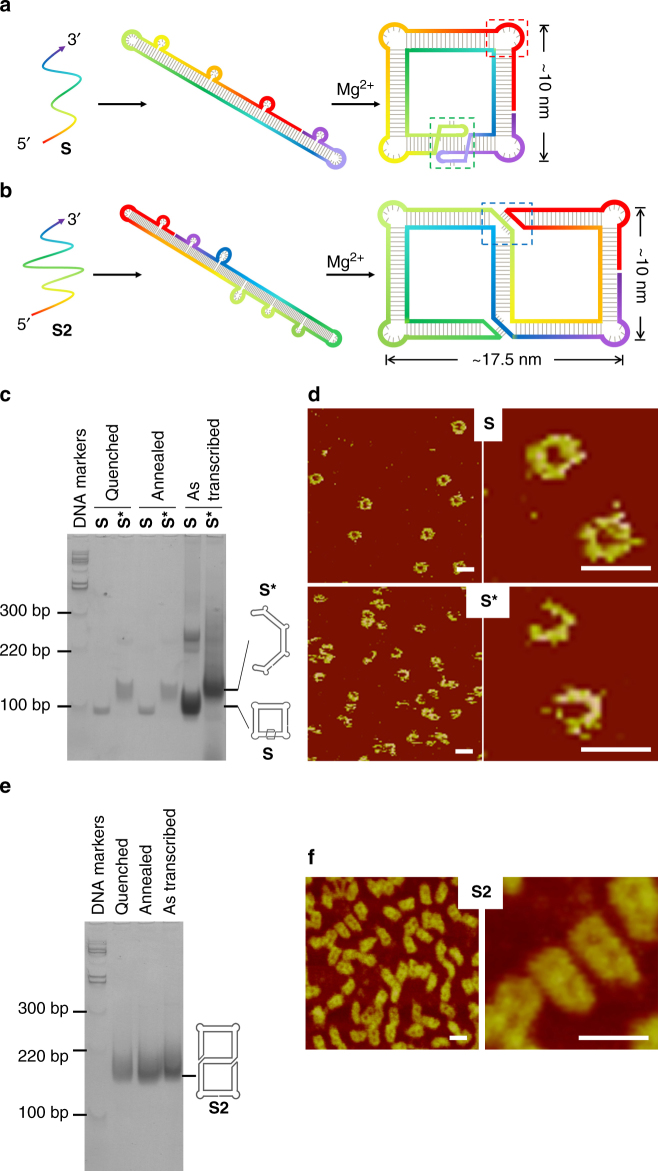
Designs, folding, and characterization of an RNA square (**S**) and an RNA double square (**S2**). **a**, **b** The molecular design and single-stranded folding pathways for **S** and **S2**, respectively. The RNA single strands are colored in a rainbow gradient from 5′ (red) to 3′ end (purple). Red, green, and blue boxes with dashed lines highlight a 90°**-**kink, a KL interaction, and a 3WL interaction, respectively. Each edge is composed of a two-turn RNA duplex. Characterization of **c**, **d** RNA square and **e**, **f** RNA double square. **c**, **e** electrophoretic analysis; **d**, **f** Atomic force microscopy (AFM) imaging. Note that S* has the same sequence as **S** except that one loop sequence is altered so that no KL interaction can form. (Scale bar: 20 nm)

We first tested this approach by the assembly of two 2D structures: a square (**S**) and a double-square (**S2**). Figure 2 shows the designs and experimental characterizations (For RNA sequences and 3D models, see Supplementary Figs 1 & 2). RNA **S** contains four 90°-kinks and a KL interacting pair, while **S2** contains four 90°-kinks and two 3WL pairs. After in vitro transcription, purified RNA molecules were thermally annealed (slowly cooled from 65 °C to 4 °C over 2 h in a neutral, Mg^2+^-containing, aqueous buffer) to promote the RNA folding. RNA molecules first folded into Mg^2+^–independent, intermediate, loose secondary structures, which then further folded into Mg^2+^–dependent, compact, tertiary structures via loop-loop (KL or 3WL) interactions. The resulting RNA square (**S**) samples were analyzed by native PAGE (Fig. 2c). A dominant sharp band is observed; suggesting that the RNA square (**S**) is fully folded. To show the critical role of the KL interaction in the folding, a control molecule (S*) is prepared, in which one loop sequence is altered so that the KL interaction is interrupted (Supplementary Fig. 1). S* migrates slightly slower than **S**, indicating that S* can fold into the open intermediate secondary structure, but not the compact tertiary structure. The formation of **S** is a unimolecular process and is expected to be kinetically fast. To prove this hypothesis, we performed a quenching experiment by plunging the RNA solutions from 65 °C onto the ice. In the native PAGE, the quenched samples (both **S** and S*) migrate identically to those annealed samples, indicating that the intramolecular folding of the designed RNA molecules is indeed a fast process. In addition, the comparison between **S** and S* molecules gives an estimation of the folding yield of **S** close to 100% as no RNA in the lane of **S** migrates like the partially folded S*. More strikingly, the RNA molecules could spontaneously fold into the designed structures cotranscriptionally. After transcription, the crude RNA samples were directly analyzed by PAGE without any purification or thermal treatment (the lane as indicated As Transcribed). The migration pattern was identical to those of purified and thermally treated samples, indicating that, as expected, the RNAs folded into the designed structures even in crude solutions without any annealing step. A similar result was also observed for **S2** (Fig. 2e). This result points to a great potential of expressing RNA nanostructures inside cells. It is worthy noting that for single-component RNA folding method, any experimental issue related to stoichiometry is avoided.

Direct visualization by AFM confirms that the RNA molecules indeed fold into the designed structures (Fig. 2d, f). In the AFM images, randomly distributed RNA particles exhibited uniform sizes. Their apparent shapes are closed and consistent with the designs: **S** particles as squares and **S2** particles as double squares. In contrast, the control molecule, S*, appears as a very different, open geometry because the KL interaction is disrupted. Please note that AFM images give a direct visualization of the RNA structures. However, discrete frameworks could be easily disrupted during sample preparation and by AFM probes, resulting in artificial structural heterogeneity. Thus, the RNA folding efficiency should not be estimated by AFM imaging of a small number of objects; instead, native PAGE of the bulk sample gives a reliable estimation.

### RNA nanoflower and tetra-square

**Fig. 3:**
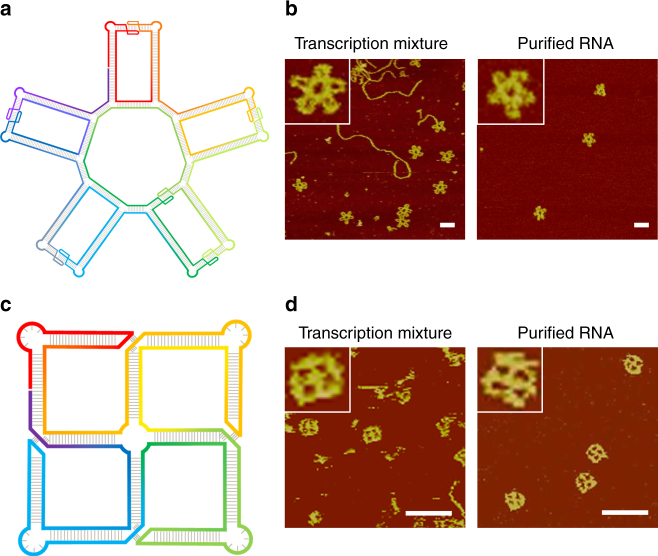
Folding of complex RNA nanostructures from single RNA strands. **a** Structural design of an RNA 5-petal flower, which contains six KL interactions, ten 90°-kinks, and ten 3WJs. **b** AFM images of the RNA flowers. Left: transcription mixture from a PCR mixture. Right: thermal annealed, purified RNA nanoflower molecule. Scale of inset: 60 nm. **c** Structural design of an RNA tetra-square (**S4**), which contains four 3WLs, four 90°-kinks, and a 4WJ. **d** AFM images of the RNA **S4**. Left: transcription mixture. Right: thermal annealed, purified RNA **S4**. Scale of inset: 25 nm. (Scale bar: 50 nm)

To demonstrate that this approach is versatile and could be used to assemble large, complex geometry, a 5-petal nanoflower and a tetra-square (**S4**) were designed (Fig. 3, Supplementary Figs 3 and 4). The RNA flower is 1571 bases long and the final structure contains five petals. The folding pathway is illustrated in Supplementary Fig. 3b. The RNA quickly folded into an intermediate, highly branched, secondary structure, which is composed of multiple duplex regions and multiple loops. Via KL interactions, the branch ends pairwisely associated with each other to form the flower petals. Note that ten 90°-kink loops, six KL interactions, and ten 3WLs taken from phi29-pRNA were integrated into the RNA strand to facilitate the desired folding. The expected five-petal-flower particles were clearly observed under AFM (Fig. 3b), confirming that the RNA indeed folded into the designed structure. In the transcription mixture from a crude PCR mixture (no purification for DNA templates and RNA), some linear structures (150–400 nm long) were observed. They were unfolded or partially folded RNA molecules, or truncated RNA molecules and DNA templates (the designed DNA template is 1591 bps or 540 nm long). In the purified RNA samples, only the 5-petal nanoflowers remained in the AFM image. Such a complicated pattern would be quite challenging for the traditional tile-based method, which would involve multiple different tiles and sophisticated inter-tile interactions, generally leading to a low assembly yield. With this ssRNA folding strategy, complicated structures could quickly self-fold with high yields. And this strategy was further confirmed by the success of the folding of an RNA tetra-square (**S4**) structure (Fig. 3c, d and Supplementary Fig. 4).

### RNA tetrahedron

**Fig. 4:**
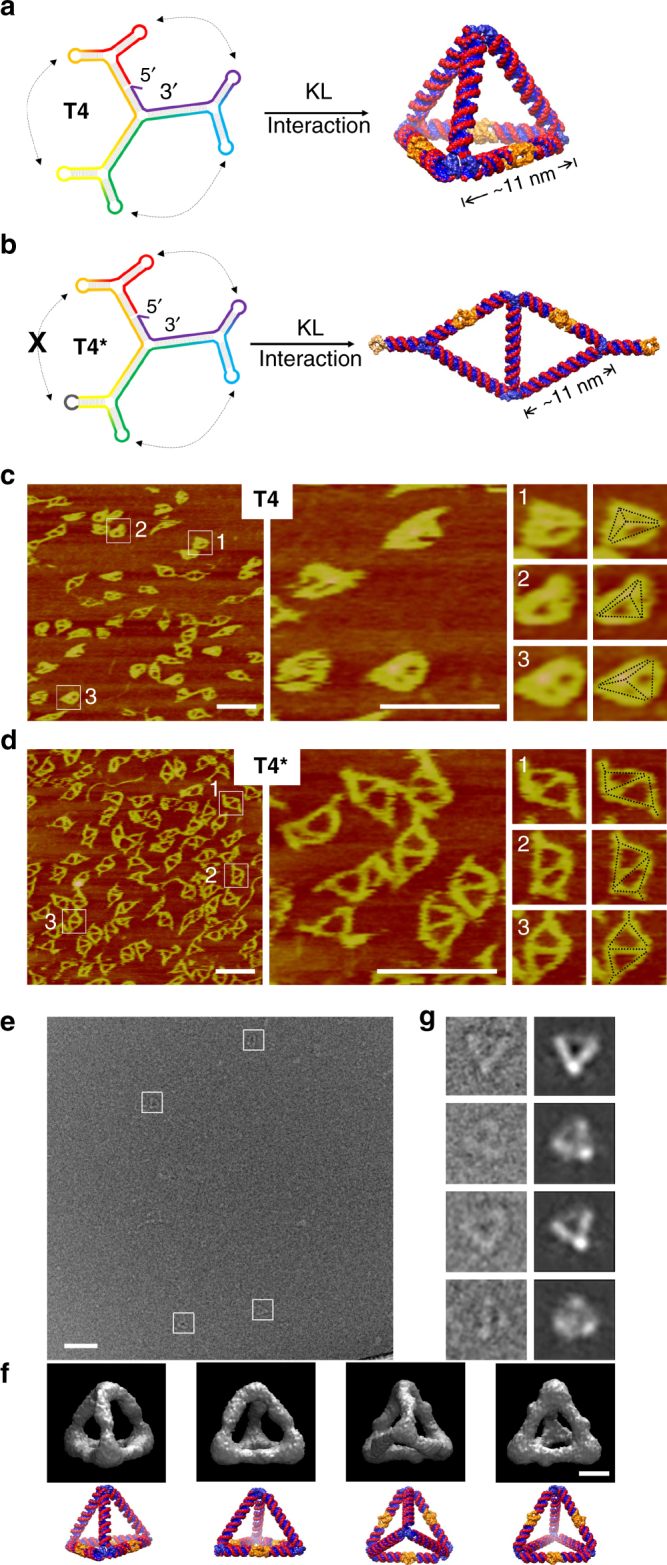
Folding of a single-stranded, 4-turn, RNA tetrahedron (**T4**). **a** Structural design of **T4**. An RNA single strand is rainbow colored from 5′ to 3′ end. It contains four 3WJs and will fold from a 2D branching structure into a 3D tetrahedron upon the three pairs of KL interactions (indicated by dashed, double-arrowed lines). Each edge is four helical turns long. Scale bar, 50 nm. **b** A control molecule T4*. One loop sequence is altered to prevent one KL interaction. Thus T4* will assemble into a flat, double-triangular shape instead of a tetrahedron. The models were built with Coot^39^ and Chimera^37^. All KLs are colored orange in the model. Scale bar, 50 nm. **c**, **d** AFM images of **T4** and T4*, respectively. For each structure, three particles are zoomed-in and fitted with corresponding shapes. **e**, **g** Cryogenic electron microscopy (cryoEM) characterization of **T4**. **e** A raw cryoEM image. Each white box indicates an individual RNA particle. Scale bar, 50 nm. **f** Four different views of the reconstructed structural model of the RNA tetrahedron (top) and corresponding views of the simulated model (bottom). Scale bar, 5 nm. **g** Pairwise comparison between raw cryoEM images of individual particles (left) and the corresponding projections (right) of the reconstructed structural model. The raw particles were selected from different images to represent views at different orientations

One important test of molecular self-assembly is to generate discrete, 3D nanostructures, which can be readily achieved by the reported strategy (Fig. 4). For demonstration, a tetrahedral structure (**T4**) is designed to fold from a 623-base-long ssRNA. It contains six edges and four vertices. All edges are four helical turns long. Three of them are standard A-form duplexes and each of the other three contains a KL interaction in the middle. Each of the vertexes is an o3WJ containing four unpaired uracils on each strand at the center to ensure sufficient out-of-plane flexibility to fold. Upon cooling, the ssRNA first folded into a three-branched structure (Fig. 4a, left), and then closed into a tetrahedral geometry via KL interactions (Fig. 4a, right). To show the importance of the KL interactions, a control molecule (T4*) was prepared, in which a pair of KL interaction was disrupted by sequence mutation. It is expected to only fold into an open, 2D, double-triangular shape instead of the compact, fully folded, 3D tetrahedron (Fig. 4b).

We have characterized the tetrahedron by native PAGE (Supplementary Fig. 6), AFM imaging (Fig. 4c, d), and cryoEM (Fig. 4e–g). PAGE analysis suggests that RNA molecules efficiently fold into the designed structures (Supplementary Fig. 6a). Each sample appears as a sharp, dominant band in the corresponding lane. The migration pattern is consistent with the expected structures. The fully-folded, compact, 3D tetrahedron (**T4**) has a higher mobility than the open, 2D, double-triangle structure (T4*). The folding is fast, and no difference has been observed between quenching and annealing for each sample. In the lane of **T4**, no RNA migrates like the partially folded T4* molecule; suggesting that all **T4** molecules are fully folded. In order to confirm the migration pattern and the folding ability, we have prepared two more RNA tetrahedrons with different sizes and their corresponding control molecules: a three-turn-edged tetrahedron (**T3** and T3*, 484 bases long, Supplementary Fig. 5) and a two-turn-edged tetrahedron (**T2** and T2*, 340 bases long, Supplementary Fig. 5). Similar migration patterns are observed (Supplementary Fig. 6b-c). AFM imaging gives more direct structural clues. **T4** samples appear compact and consistent with a collapsed tetrahedral geometry (Fig. 4c), while T4* samples exhibit clear double-triangle structures (Fig. 4d and Supplementary Fig. 7). Please note that some **T4** structures are disturbed during AFM imaging.

CryoEM study revealed the native, 3D, tetrahedral structure of **T4**. In raw images of the **T4** sample (Fig. 4e), uniform-sized particles could be easily identified (indicated with white boxes). Their shapes resembled the 2D projections of a tetrahedron. From the observed individual particles, a technique of single-particle 3D reconstruction was applied and resulted in a structural model of a tetrahedron at a resolution of 4.1 nm (Fig. 4f). To verify the reconstructed model, two comparisons were performed: the computed 2D projections with individual raw particles (Fig. 4g) and with class averages of raw particles with similar views (see Supplementary Fig. 8). Clear similarities in the pairwise comparisons verified the reconstructed model. Note that the six struts in the model were not the same.

### In vivo production of RNA nanostructures

**Fig. 5:**
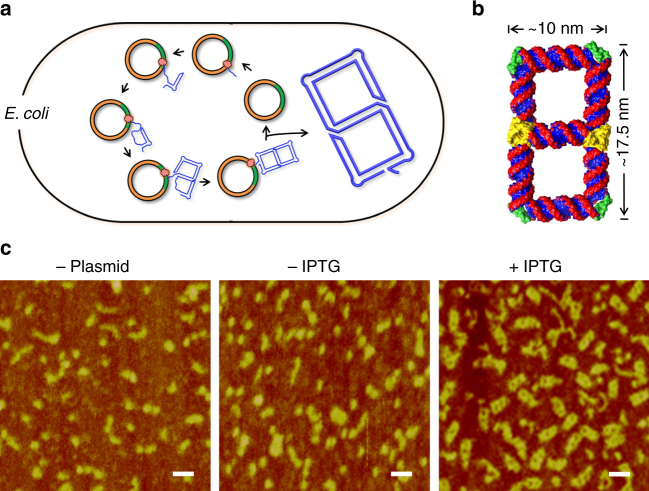
Cloning and in vivo expression of RNA nanostructures. **a** Overall scheme. An expression vector (pET23a, orange) carrying an **S2**-coding DNA sequence (green) is transformed into *E. coli* cells. Upon IPTG induction, the **S2** gene gets transcribed into RNAs, which self-fold into the designed double-square structure in the cell. **b** Structural model of the **S2** structure. **c** AFM imaging of cell lysates from *E. coli* without the expression vector (- Plasmid), or with the vector but without **S2** gene expression induction (- IPTG), or with **S2** gene expression ( + IPTG). (Scale bar: 20 nm)

The most promising feature of the current approach is that it is compatible with in vivo expression of the designed RNA nanostructures. In our design, each RNA nanostructure contains one single strand. Therefore, an RNA strand, in the same way as peptides fold into proteins, can spontaneously fold into the designed structure under the physiological condition during in vivo transcription. To demonstrate this capability, we have cloned and expressed the double square structure (**S2**) in *E. coli* (Fig. 5). Briefly, the **S2**-coding DNA sequence is inserted into a pET23a plasmid, a bacterial expression vector, under the control of a T7 promoter (Supplementary Fig. 9a). The resulting recombinant plasmid is then transformed into BL21 (DE3) *E. coli* cells and the RNA transcription is induced by isopropyl β-d-1-thiogalactopyranoside (IPTG). Then the cells were lysed by phenol or sonication. Without any further manipulation, the aqueous solution of the total cell lysates was directly examined by both PAGE and AFM imaging. On native PAGE, the desired RNA molecule from *E. coli* migrates the same as the in vitro prepared **S2** sample (Supplementary Fig. 9c), suggesting that the RNA from the cells is well folded as the expected double-squared structure. This is further confirmed by AFM imaging (Fig. 5c and Supplementary Fig. 9d). In the raw cell lysate after expression induction (addition of IPTG), abundant double-square shaped particles are clearly identified, which are identical to the **S2** sample prepared from in vitro transcribed RNA (Fig. 5c: samples from phenol lysis method; Supplementary Fig. 9d: samples from sonication method). In contrast, no double-square shaped particles are observed from the cells without the **S2** gene or the cells without expression induction. Based on the band intensities in the PAGE graph (Supplementary Fig. 9b), we can estimate that **S2** molecule accounts for roughly 11.2% of all RNAs in the cell; suggesting that in vivo expression is potentially an efficient way to produce RNA nanostructures. Similarly, we can in vivo express the tetra-square (**S4**) structure (Supplementary Fig. 10). It seems that the RNA folding is efficient, but the RNA transcription yield is low or the RNA is not stable in the cell. In the future, it might be worthy using natural RNAs, e.g., tRNA or 5 S rRNA, as scaffolds to improve the RNA production yields of longer and complex strands.^14, 15^

## Discussion

In vivo production of nucleic acid (DNA/RNA) nanostructures is a major challenging to the field of nucleic acid nanotechnology and has attracted significant efforts. The original idea was first proposed by Nadrian Seeman in 1997.^28^ But the complicated topology associated with that design prevented it from experimental realization. The first experimental progress was elegantly done by Shih et al. in 2004.^29^ They assembled an octahedron from a long enzymatically generated DNA single strand and a few helper strands. In 2006, Paul Rothemund developed a powerful and generally applicable strategy, so-called DNA origami. It folded long, biologically produced, M13 bacteriophage genome by hundreds of chemically synthesized, short DNA strands into designed nanostructures.^5^ In 2008, Lin et al. cleverly used bacteria to produce simple DNA nanostructures, such as a DNA four way junction.^18^ In 2011, Delebecque et al. produced RNA molecules, which further associated with each other to organize chemical reactions in *E. coli.*^12^ In 2011, Afonin et al. reported in vitro, co-transcriptional assembly of multi-stranded RNA complexes.^13^ In 2013, Ducani et al. enzymatically, either in vitro or in vivo, produced ssDNA, which were released with restriction enzymes in test-tubes and used to assemble DNA nanostructures with thermal annealing.^30^ In 2014, Geary et al. developed a strategy to in vitro fold long RNA strands into nanostructures.^9^ This strategy is closely related to our work. In 2016, Elbaz et al. took advantage of reverse transcriptase to in vivo produce ssDNAs, which could in vivo assemble into multi-stranded DNA nanostructures.^31^ In 2017, Hendrik and coworkers used bacteria to cost-effectively and at large-scale produce single DNA strands, which in vitro self-assembled into multi-stranded, DNA nanostructures upon thermal annealing.^32^ In 2017, Yan and coworkers enzymatically produced DNA or RNA single strands, which, upon thermal annealing, in vitro folded into designed nanostructures without topological tangling.^33^

In conclusion, we have developed a programmable ssRNA folding strategy for the preparation of complex nanostructures. Each nanostructure is primarily composed of RNA duplexes and is folded from one long ssRNA. Thus, the formation of the nanostructures is independent of stoichiometry and RNA concentration. This strategy allows cloning and expression of RNA nanostructures inside of cells in the same way as those of recombinant proteins; pointing to a cost-effective way for large-scale production of nucleic acid nanostructures. We expect this strategy would facilitate a wide range of in vivo biomedical applications of RNA nanotechnology, such as integrating multiple functional RNA moieties to regulate cellular processes.

## Methods

### Oligonucleotides

Double-stranded DNA templates were purchased from IDT, Inc. Polymerase chain reaction was applied to amplify DNA duplexes into large amount. The sequence of DNA templates can be found in Table 1.

**Table 1:** Sequence of DNA templates

S	5′-**TTCTAATACGACTCACTATA**CCGTGTACTCGGAGGAACTACTCATAT TCGAGCGTCGGAGGAACTACTGCAGTGGCTCGTTATTCGGAACTACTATGGTGAAGGAGGCACGCCATAGCCGAATAACGAGCCATTGCAGCCTCCGACGCTCGAATGTGAGCCTCCGAGTACACGGGCTGAGCCACGTGAAGCCTCCACGCGTGGAACTACTCAGCAGTGCTAAGAATCTA-3′
S*	Identical to S except the underlined sequence was replaced by AAAAAAAAA
S2	5′-**TTCTAATACGACTCACTATA**GACTCGGAGGAACTACTCACATTC GAGCGTTGATGGAGGTCAACGCTCGAATGTGAGCCTCTGAGTCCTGTCGATCAGCCTGGTCTAGCATCTATCAGCCCAGATTCAACTATTGGGGATACTCCCTGAATCGGATCACGCACGGAACTACTGTAGTGCTTCGTTAGTCGGAACTACTGCTCTAGATAGGTTGATGTCGGTCAACCTATCTAGAGCAGCCGACTGACGAAGCATTACAGCCGTGTGTGATCCGATTCAGCCCAGTAGTTGAATCTGGGGATACGACCTGATAGGTGCTAGGCCAGGAACTACTGATTGACAGGCAGTGCTAAGAATCTA-3′
FL	5′-**TTCTAATACGACTCACTATA**CTCTAGAAGACCGAGGCTGGCTATAT GAGGAACTACTCGGTGAAGAGGAGACGCCGAGCCTCATGTAGCCAGTCTCGGTCTCCGTCGTCAGACACTCGGGTGTGTGTCGTTGCGTCGACGGGTTGTGTGTCCTGTCTTGGCAGGTCTGGACCGGGAGCGCTTGAGCTCAGCCATTTGAACTCCTCACGAATGGAACTACTGAGTTCAAGTGCTCCTGGTCCGGACCTGTCAAGACGGGACACTTTGTAGGGTGGTGTTTCACGGGACATCACCTGTGGAGGGTTATCGGTACTCTGGGCACGGAACTACTGCATGAATCGAGCACGTGCAGCCGTGCTCAGAGTGCCGATGACCCTCTACAGGTGGTGTCCCGTGTGTAACGTCTCGAAGAGGAGGTGTGTCAAGTAGGCACAGCTGAGGTTCGAACGGACTTCGGCTCCCAGCCTGGTGAAGCTCGAACGCCAGGAACTACTGGGAGTCGAAGTCTGTTCGAATCTCAGTTGTGCCTGCTTGACTTTGTCAGGGAGTGTTTCAGTAGTAACGGGCCGACTAACTGGAGCGTGGGAAGATCATTGGAACTACTAGCTGAAACGGCAACGGCTAGCCAATGGTCTTCCTACGCTCTAGTTGGTCGGTCCGTTGCTACTGTGTGTTTGAACACTGGACGAACACTACTCCCTGATCACCTTCTCTTTGAGATGTTACTACCACCCTATCAACCTGTCGGCGCAATGACACTACCCTGGGATCAGTCTGATCTATCGTGACGACTAGAGCTCGATCAACAGGCTATTCGGTCAAGACTACTCTCAGATCACGGTGAACCAGTGACGCCGTGTGTCGAGTCAACTGCGTTGCGTCCGTATCACTACCTCTTGGAGAGCCCAGTGAATGCCGTACGCTGGGAACTACTCTCCGAGAGGTGGTGATGCGGATGCAACGTAGTTGGCTCGACTTTGTCTGAGAGTGTTTCAGCATGCGTCAGCTCTGTGGTGAATTCAGCTAGCACTGTCAGGAACTACTCAGTGAAGATAGGACGCTGAGCCTGACGGTGCTAGTTGAATTCATCACAGGGCTGATGCATGCTGTGTCTTGGCCGAGTAGCTTGTTGTGTCGGTGTCACCGTATGGACATCTGTCTTCAGCATTGGCCCTAGCCGGCTGAACCTATCACGGCCGGAACTACTAGGGTCAATGCTGGAGACAGGTGTCCATGCGGTGGCACCGACTTTGTCGAGCTCTGTTTCACTAGGAGGATCCGAGCATTCTTCACTGGGGTTCTCGTGGTGGAACTACTCACCGAAGCACGTACGGTGAGCCACCATGAGAACTCCAGTGGAGAATGTTCGGATTCTCCTAGTGTGTCGTCGCGATGGATCGGACTGTGTGCCTGGACGCAGTCCCTTACGCTCTGCTAGGGTGCTACTTAGCCAAGTGAAACGTGCACGCTTGGAACTACTAAGTGGCACCTTAGCGGAGCGTGAGGGATTGCGTTCAGGCACTTTGTCCCAGGGTGTTTCACCCGGGTGTCTGATGACGGACTAGCATAACCCCTTGGGGCCTCTAAACGGG-3′
S4	5′-**TTCTAATACGACTCACTATA**GG*TCTAGA*GGTGAGTGTGTAACTACG GTCTAGTCGTGGAGGAACTGGTCGTAGTTCCTCCGCGACTAGGCCGACACACTCACCCATCACTCCAGCCACTTCCAGGGAAACTCCGGCTTCGTGTGATCTCGACATTCTGGGGATACGACCTGGTTACCCTCCTAGACTATCAACTAGGTGGGAGTGAATGCTGTTCTGGGAGTAGAACAGCGTTCACTCTCACCGATAGTCTGGGAGGGTAATCAGCCCAGAGTGTCGAGATTACACGTTCGTACAGACACGTCTGAGTGGGGATACTCCCTGAACTCAGGTGCTTGAGCTCAACTAGGAGTTGAGTCTTCTCAACGTGTTGCTACGTTGAGAAGATTCAACTCCGAGTTCAAGCACCTGGGTTCAGCCCACTCAGGCGTGTCTGTGCGTTGTGAGTATCGACAGGATAGAGGGATAGCAACGTGCAGTGGAGAACATTACACAACTAGTGTCAGAGCTAGCAGGTCGTGAGCGTACGACCTGCTGGCTCTGACACGTGTGATGTTCTCCATTGCACGCCTCTGTCCTGTCGATGCTCACTTGCTGGAGTTTCTCTGGAAGTGGGATACGCTCTGGAGTGATG*CCTTGG*GGCCTCTAAACGGG**-**3′
T4	5′-**TTCTAATACGACTCACTATA**GACGTGTAGTTGTCTGTTACCTTTTTA
	GCGTGTAGATAGTCGCTACAAAAGCGGTATGTAGCGACTATCTACGCGCTTTTTAATCAGACGCAAGTTTATCAACACCTGAGATAGACTTGCGTTTGATTTTTTAGGTAACAGACAACTACATGTCCCCAATCTAGTCTCAGTCCGGATTTTGAGGATGAGCACATTTGCGCGTATGTGGTCCGAAGGTCATGGCATTTTTCCGTACATCAACTTAGGTGCAACAGGTGAGCATCTAAGTTGATGTACGGATTTTGTGCGTCGCTCGACGGCCGAAACGGCAACGGTCGTCGAGCGATGCACTTTTTGCCATGACCTTCGGACCACATACGCGCGAATGTGCTTATCCTCTTTTTCATGCGATGGCTCGGCGGCTCTTGGTACATTCTGGCTCGACTTTTTTAGGCTTCTCTGTCACGGATGCAATGCCGTAGCATTCGTGACAGAGAAGCCTTTTTAGGTTAACTACACGGATATAAACCGCTAATATCTGTGTAGTTAGCCTTTTTAAGTCGAGCCAGAATGTACCAAGAGCCGTCGAGCCATTGCATGATTTTTCCGGATTGAGACTGGATTGGGGCAGTGCTAAGAATCTA -3′
T4*	Identical to T4 except the underlined sequence was replaced by AATTTTTTA
T3	5′-**TTCTAATACGACTCACTATA**GACGTGTAGTCGCCTGTTACCTTTTTA
	GCGGCTACAAAAGCGGTATGTAGCCGCTTTTTAATCAGACGCAAGTTTATCAACACCTGAGATAAACTTGCGTCTGATTTTTTAGGTAACAGGCGACTACACGTCCCCAATCCGGATTTTGAGGATGAGCACATTCGCGCGTAGGTCATGGCATTTTTCCGTACTGCAACAGGTGAGCAGTACGGATTTTGTGCATCGCTCGACGGCCGAAACGGCAACGGCCGTCGAGCGATGCACTTTTTGCCATGACCTACGCGCGAATGTGCTCATCCTCTTTTTCATGCAATGGCTCGGTAATTCTGGCTCGACTTTTTTAGGCTAATGCAATGCCGTAGCATTAGCCTTTTTAGGCTAACTACACGGATATAAACCGCTAATATCCGTGTAGTTAGCCTTTTTAAGTCGAGCCAGAATTACCGAGCCATTGCGTGATTTTTCTGGGTTGGG-3′
T3*	Identical to T3 except the underlined sequence was replaced by AATTTTTTA
T2	5′-**TTCTAATACGACTCACTATA**GGCTACTGTTTATAGCGAGGCCGTG ATTCGCTTTTTAAAAGTCATAATGCCGTAATGACTTTTTTTTTCGAGCCGAATCGCCAACGGCTCGAATTTAAGCGAATCACGGCCTCGCTATTTTAATCGATGCATCTGACTGCTCTTTTAGACGCUCGAATGGCGAACGGGCGTCTTTTAGTACGTCCAAGTCCACAGGACGTACTTTTAGAGCAGTCAGATGCATCGATTTTTCAGTAGCCGCATTGUATACTGCTTTCTGAGTACCAAGTGGACAGGTACTTAGTTTCGGTGCGGCAAACGGCAAGCCGCACCGTTTGCAGTATGCAGTGCTAAGAATCTA-3′
T2*	Identical to T2 except the underlined sequence was replaced by AATTTTTTA
S2’	5′-**TTCTAATACGACTCACTATA**C*TCTAGA*ACAGGCGGAGGAACTACTC ACGTTCCAGCGTTCATGGAGGTGAACGCTGGAATGTGAGCCTCTGCCTGCTCCTGATCAGCCACGTGTGACATCTAAGAGCCCATCCTCACCAGTAGGGGATACTCCCTGAATCGGAACATGGACGGAACTACTGTAGTGCTTGGTTAGTCGGAACTACTGCACGAGGTAGGTTGATGTCGGTCAACCTATCTCGTGCAGCCGACTGACCAAGCATTACAGCCGTCCGTGTTCCGATTCAGCCCTGCTGGTGAGGATGGGGATACGACCTCTTAGGTGTCACGCGTGGAACTACTGATTAGGAGCTAGCATAACC*CCTTGG*GGCCTCTAAACGGG-3′

T7 promoter sequences are highlighted in bold

Two restriction enzyme sites, XbaI and StyI are formatted in italics (for in vivo double-square S2′ and for tetra-square S4)

(FL: RNA nanoflower, S2′: double-square for in vivo expression)

Sequence of PCR primers:

P1: 5′-TTCTAATACGACTCACTATA-3′

P2: 5′-TAGATTCTTAGCACTGC-3′

P3: 5′-CCCGTTTAGAGGCC-3′

P4: 5′-CGTTATTGTACCCAACCCAGAAAAATCAC-3′

S/S1*/S2/T2/T2*/T4/T4*: P1 + P2; FL/S2’/S4: P1 + P3; T3/T3*: P1 + P4.

### DNA sequence design

When designing RNA sequences, we take two parts into consideration. (i) The single stranded loops in the RNA secondary structures, including the 90°-kink loop, KLs, pRNA interacting loops. They are critical regions and determine the long-range interactions and local conformations. For the 5-nt-long, 90°-kink loop, the sequence is strictly taken from the HCV IRES domain II.^27^ For KLs and pRNA loops, the exact sequence is not important, but sequence complementarities are ensured for the interacting pairs. (ii) The remaining duplex regions. They provide all the struts in the structures and do not involve long-range interactions. Their sequences are randomly generated by a computer software Tiamat.^34^ Roughly 10% of the Watson-Crick basepairs (A–T and G–C) are replaced by G–U pairs to facilitate PCR amplification of the DNA templates. Such G–U pairs are evenly distributed along the duplex regions. Finally, the RNA sequences are checked by a computer software Mfold^35^ to make sure that the RNA will fold into the designed secondary structures. If there are any undesired, strong secondary structures, the sequence will be manually altered to avoid such structures.

### Polymerase chain reaction

DNA double-stranded template was amplified by polymerase chain reaction using Taq DNA polymerase kit (New England Biolabs Inc.). 4–10 ng DNA template, 200 µM dNTPs, 200 nM of forward and reverse primers were dissolved in 100 µL standard Taq reaction buffer. Then 0.5 µL Taq DNA polymerase was added to the system. The solution was initially denatured for 30 s at 95 °C, and started 30 cycles at 95 °C for 30 s, 50 °C for 1 min, and 68 °C for 2–3 min. Then kept the final extension at 68 °C for another 5 min. PCR product was stored at 4 °C for short time storage and at −20 °C for long terms.

### In vitro RNA preparation

RNA molecules were synthesized from in vitro transcription using T7 RNA polymerase (AmpliScribe T7-Flash transcription kit; Epicenter, Inc.). Corresponding DNA template was added, and the experiment was conducted by following the manufacturer-recommended protocol.

### Formation of ssRNA structures

ssRNA was prepared in TAE/Mg^2+^ buffer and annealed by different processes: Quenched---65 °C/5 min, 0 °C/5 min; Regular anneal---65 °C/5 min, 50 °C/30 min, 37 °C/30 min, 22 °C/30 min, and 4 °C/30 min. TAE/Mg^2+^ buffer contained 40 mM Tris base (pH 8.0), 20 mM acetic acid, 2 mM EDTA, and 12.5 mM magnesium acetate.

### Denaturing PAGE

5% denaturing PAGE gel was prepared with the 19:1 acrylamide/bisacrylamide solution, 8 M urea, and TBE buffer, containing 89 mM Tris base (pH 8.0), 89 mM boric acid, and 2 mM EDTA. The gel was run at 55 °C for around 1.5 h at 650 V on Hoefer SE 600 electrophoresis system and was stained with ethidium bromide (Sigma). The major band was cut under UV light and eluted out.

### Native PAGE

4% native PAGE gel was prepared with 19:1 acrylamide//bisacrylamide gel and TAE/Mg^2+^ buffer. The gel was run at 4 °C. Then stained with Stains-All (Sigma) and scanned by an HP scanner (Scanjet 4070 Photosmart).

### Agarose gel analysis

Samples were loaded into 1% agarose gel and run in TBE buffer on a FB-SB-710 electrophoresis unit (FisherBiotech) at room temperature under constant voltage of 80 V. The gel was stained in EB and illuminated under UV light. Images were taken by cell phone.

### AFM imaging

Mica surface (Ted Pella, Inc.) was pre-treated with poly-L-lysine (0.1% w/v, Ted Pella, Inc.) to increase the adsorption of small RNA particles. 20 µL poly-l-lysine (5 µg/ml) was added onto freshly cleaved mica substrate and was incubated for 30 s. Then the surface was washed by 100 µL H_2_O and dried by compressed air. Annealed RNA solution was diluted to 50 nM and a drop of 5 µL was deposited onto pre-treated mica and incubated for 2 min. 20 µL TAE/Mg^2+^ buffer was further added. Imaging was performed in a fluid cell under tapping mode on a Multimode AFM (MMAFM 2, Veeco) with SNL-10 probe (Veeco, Inc.).

### Cryo-EM imaging

3 μL RNA solution (50 nM) was deposited on the Lacey Carbon-only grid (300 mesh Cu, Ted Pella, Inc.). The grid was blotted with two pieces of filter paper (Whatman, 1001-055) and was immediately flash frozen in liquid nitrogen-cooled liquid ethane. Images were taken on FEI Titan Krios with accelerating voltage of 300 kV under 90 e^−^/Ǻ^2^ dose condition at under-defocus 8 μm to enhance image contrast.

### Single particle reconstruction

The single particle reconstruction was carried out using computer software EMAN 2.^36^ Totally, 470 particles were selected to go through reconstruction process using EMAN2 reconstruction package, following the standard protocol. (Wiki at: http://blake.bcm.edu). A general summary of the steps includes: (1) Import and convert CCD micrographs; (2) Pick typical particles; (3) CTF determination; (4) Remove bad particles and build particle sets; (5) Make 2D reference-free class-averages; (6) Build an initial 3D model; (7) 3D high resolution refinement; (8) Evaluate the resolution of 3D map. About 470 randomly selected particles were used to build 12 class averages, which were used to generate initial models under different symmetries, such as C3 symmetry, TET symmetry or without symmetry (see Supplementary Fig. 8). Totally around 360 particles were used for 3D refinements of the RNA tetrahedron. The refinement was carried out with a 2° angle interval under C3 symmetry. A projection matching algorithm was applied for the determination of the center and orientation of raw particles in iterative refinement. After refinement, the class-averages of raw particles and the 2D projections of 3D model were generated in two files but paired in order. The comparisons shown in the result were manually selected to present the distinct and comprehensive orientations. The resolution of resulted structural density map was determined to be at 4.1 nm. Final reconstruction results were visualized by UCSF Chimera software^37^.

### Recombinant plasmid preparation

Double-stranded DNA template and pET23a plasmid (GenScript Inc.) were double-digested by XbaI and StyI-HF restriction enzymes (New England BioLabs Inc.) by following the manufacturer-recommended protocol. The reaction was performed in 100 µL CutSmart^®^ buffer at 37 °C for 2 h, and 20 units of each enzyme were added. After digestion, DNA was recovered by DNA clean & concentrator columns (Zymo Research Corp.) to get rid of proteins. Then the template and plasmid were ligated by T4 DNA ligase (New England BioLabs Inc.), and were incubated overnight at 16 °C.

### Plasmid cloning and bacteria culture

Ligation product was combined with XL1-Blue competent *Escherichia coli* (*E. coli*) cells for transformation. The mixture was kept on ice for 30 min, and then heat to 42 °C for 90 s and put on ice for 2 min. Spread the transformed bacteria on Lysogeny Broth (LB, 100 µg/ml ampicillin was added) (RPI. Corp.) plates and incubate overnight at 37 °C. After the colonies growing into desired size, inoculate a colony into an individual aliquot of 6 ml LB/ampicillin liquid medium and shake overnight at 37 °C. 1.5 ml bacteria solution was taken out into another 6 ml fresh LB/ampicillin liquid medium and kept shaking at 37 °C until UV absorbance was around 0.5 OD at 600 nm, measuring by a UV/Vis spectrophotometer (Beckman coulter, DU520). Plasmid was extracted using plasmid isolation kit (Zymo Research Corp.). The purified plasmid was characterized by restriction enzyme digestion and gel analysis to confirm the correct insert size.

### In vivo RNA expression induction

The recombinant plasmid was transformed into BL21 Star^TM^ (DE3) competent E.coli cells (Life Technologies Corp.) for RNA expression, following the manufacturer’s protocol. The cell culture process was the same as described above. When UV absorbance was around 0.5 OD at 600 nm, IPTG (1 mM) was added for induction and the solution was shaken at 37 °C for 3 h.

### Native extraction of RNA from bacteria

After induction, centrifuge 1 ml bacteria culture solution in a 1.5 ml centrifuge tube and remove the suspension. Re-suspend the pellet in 100 µL Buffer L, containing 10 mM Tris-HCl (pH 7.4) and 10 mM Mg(OAc)_2_. Then destroy the bacterial membrane by adding 100 µL phenol solution (Sigma).^38^ Pipet out the aqueous layer and directly deposit it into native PAGE gel or scan under AFM. To prepare samples for denaturing PAGE gel or gel purification, 10 µL NaOAc (3 M, pH 5.2) and 200 µL ethanol were added to 100 µL aqueous layer, followed by an ethanol participation in dry ice to get rid of salts. Alternatively, the cell pellet was re-suspended in 15 ml Buffer L and sit in an ice-water bath. Cells were lysed by sonication (without phenol) with Branson Digital Sonifier (10% amplitude). Sonicating for 5 s and stop 5 s; and repeat over until total of 10 min. 1 ml lysates were centrifuged at 16,000×*g* for 30 min to remove the cell debris and the upper layer was diluted 40 times with TAE/Mg^2+^ buffer for AFM imaging.

### Data availability

Data supporting the findings of this study are available within the article and its Supplementary Information Files and from the corresponding author upon reasonable request.

## Electronic supplementary material

Supplementary Information
