# Supplementary material for: In vivo production of RNA nanostructures via programmed folding of single-stranded RNAs
Source: Nat Commun. 2018 Jun 6;9:2196. doi: 10.1038/s41467-018-04652-4 (PMC5989258; doi:10.1038/s41467-018-04652-4)

In Vivo Production of RNA Nanostructures via Programmed Folding of Single-Stranded RNAs

Mo Li[1], Mengxi Zheng[1], Siyu Wu[1], Cheng Tian[1], Di Liu[2], Yossi Weizmann[2], Wen Jiang[3], Guansong Wang[4*], and Chengde Mao[1*]

Supplementary Information

Supplementary Figure 1. RNA sequences for **S** (209 nts) and the control molecule **S***. Below is a 3D structural model. Note that the single-stranded tails at 3' end are designed to facilitate PCR amplification of the DNA templates.

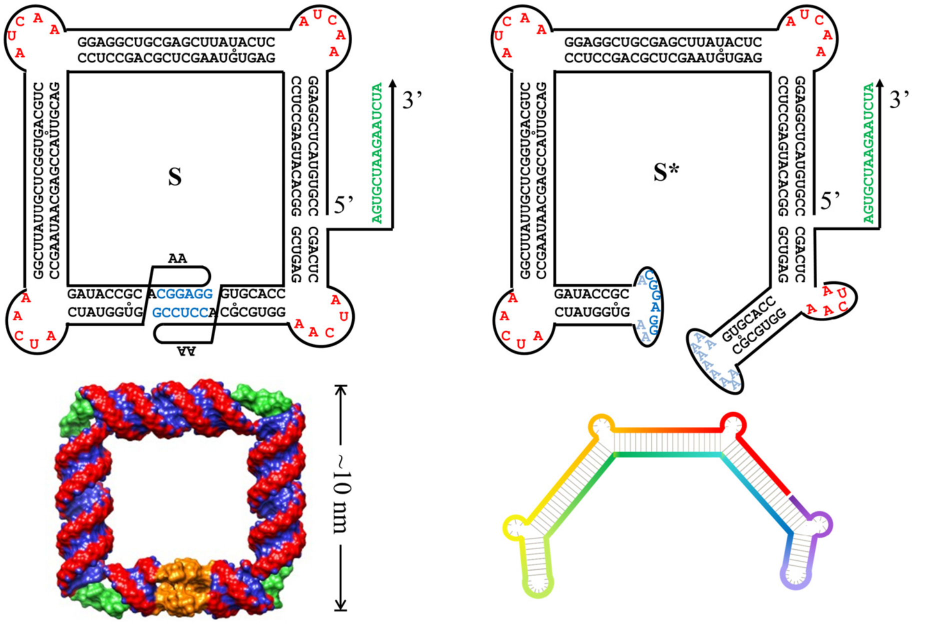

Supplementary Figure 2. RNA sequence for **S2** (339 nts). Below is a 3D structural model.

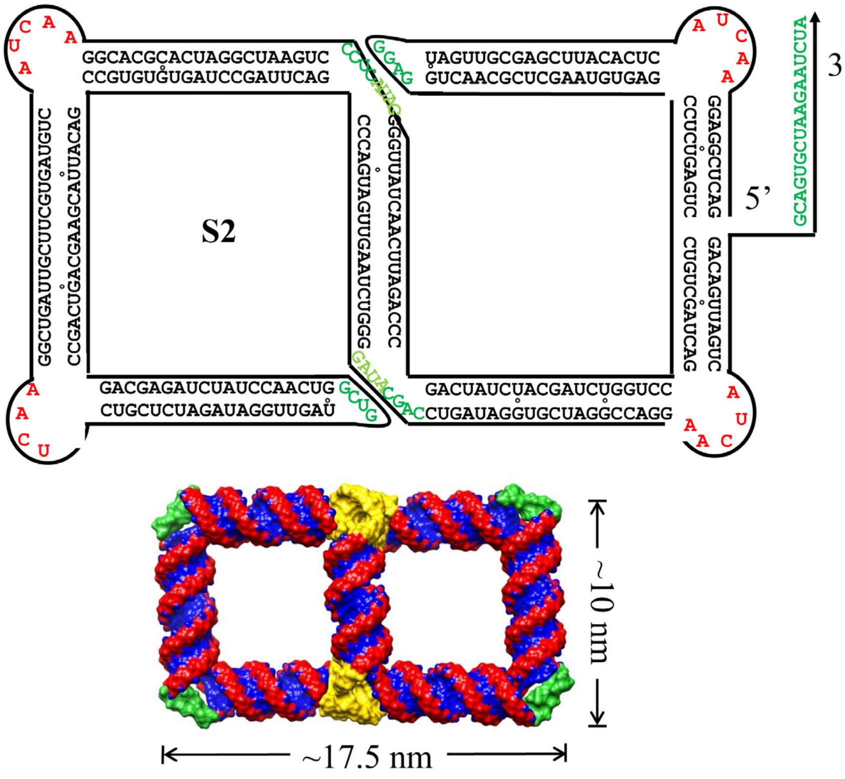

Supplementary Figure 3. RNA nanoflower. (a) RNA sequence (1571 nts). (b) Assembly pathway. The corresponding KL interactions are indicated by dashed two-way arrow lines. (c) a 3D structural model. (d) Electrophoretic analysis. (e) AFM images. Some line structures (presumably double-stranded DNA templates) were observed in the sample of transcription mixture (Scale bar 200nm), but not in the purified RNA samples (Scale bar 500nm).

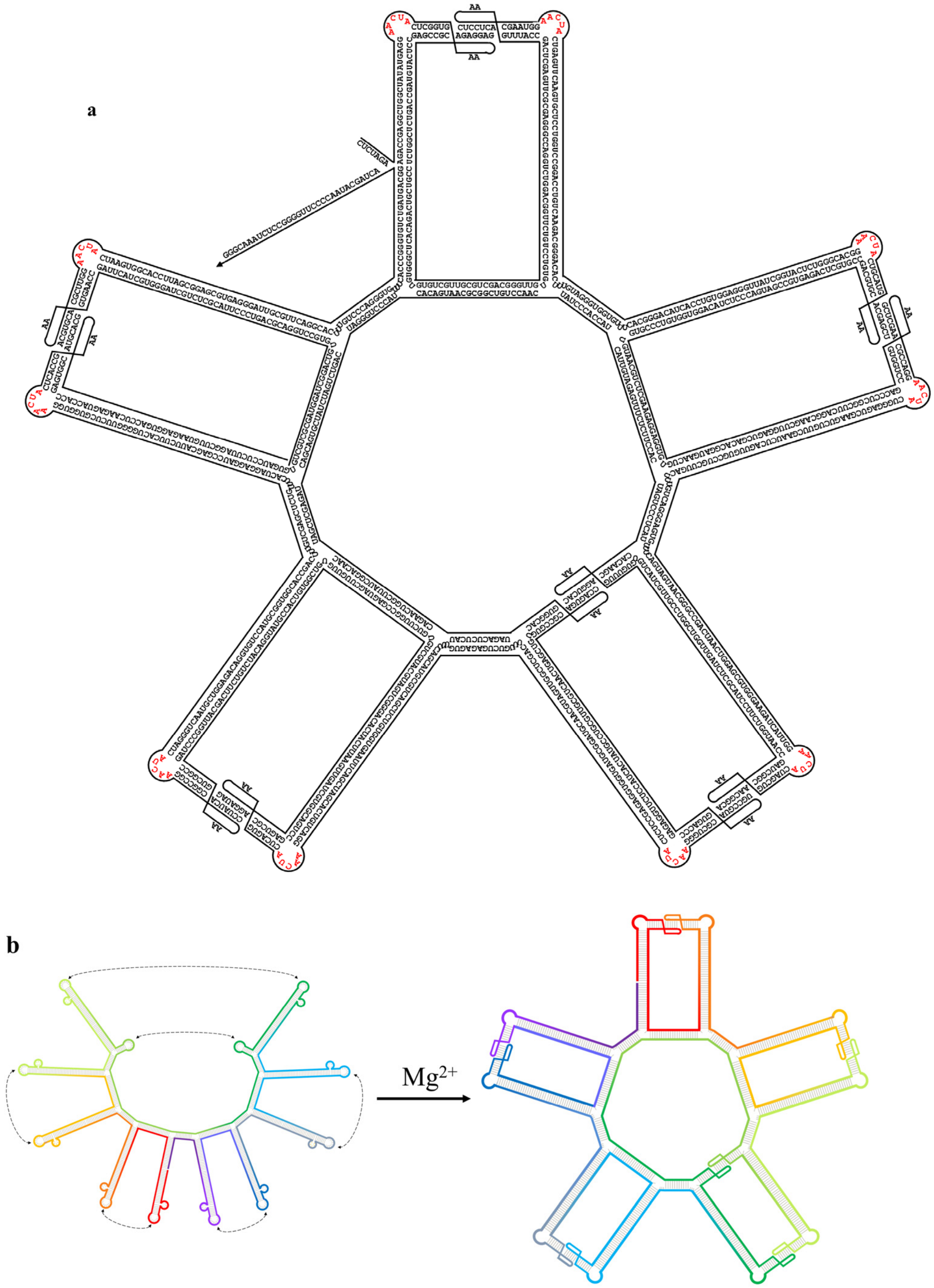

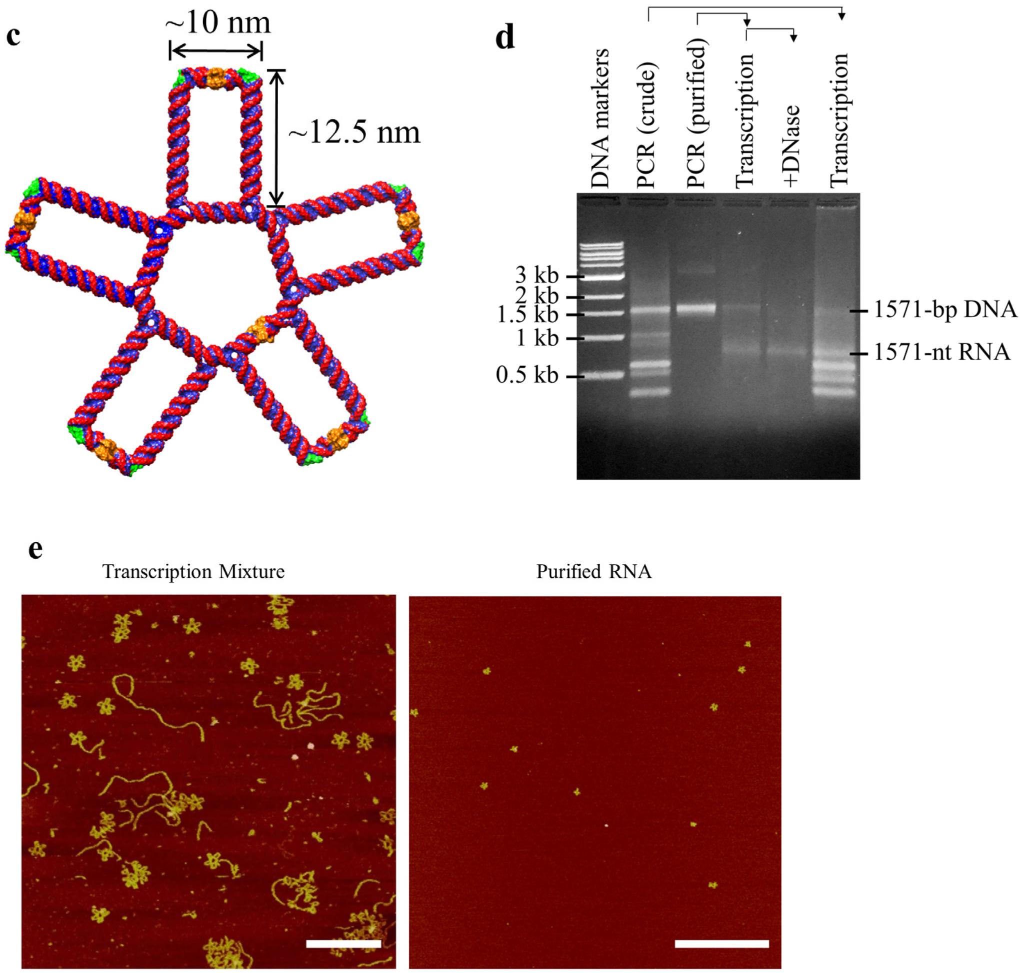
c
~10 nm
~12.5 nm
d
DNA markers
PCR (crude)
PCR (purified)
Transcription
+DNase
Transcription
3 kb
2 kb
1.5 kb
1 kb
0.5 kb
1571-bp DNA
1571-nt RNA
e
Transcription Mixture
Purified RNA

Supplementary Figure 4. RNA tetra-square (**S4**). (a) RNA sequence. (b) Assembly pathway. The corresponding KL interactions are indicated by dashed two-way arrow lines. (c) a 3D structural model. (d) AFM images. Some partial structures (presumably from RNA fragments) were observed in the sample of transcription mixture, but much less the purified RNA samples. (Scale Bar: 200 nm)

a

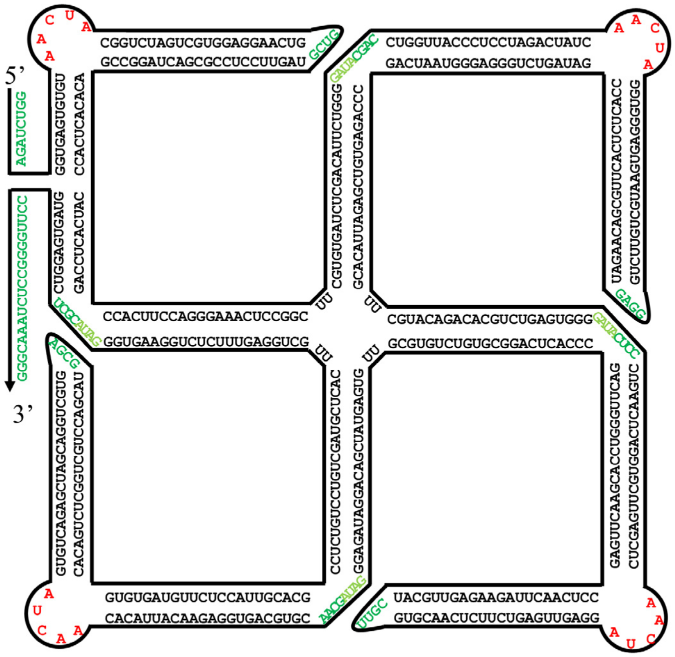

b

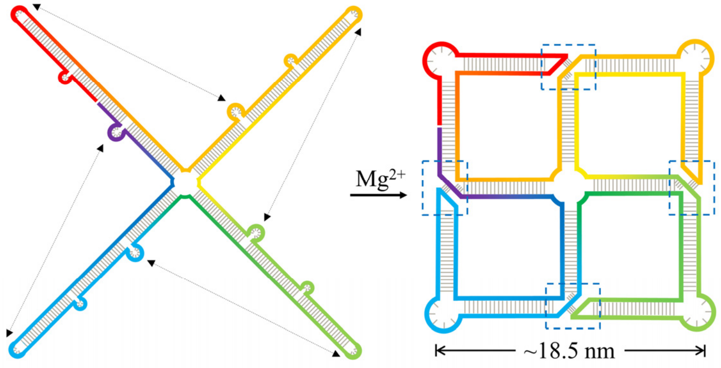

c

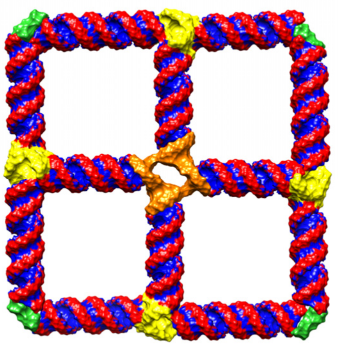

d

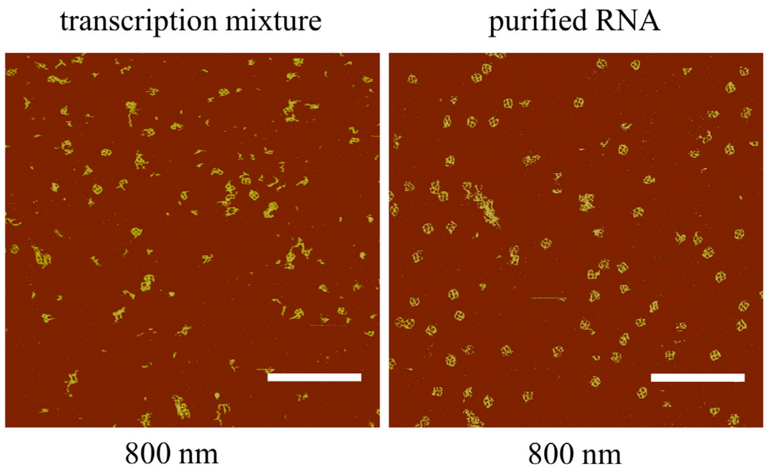

Supplementary Figure 5. RNA tetrahedron secondary structures.

T4

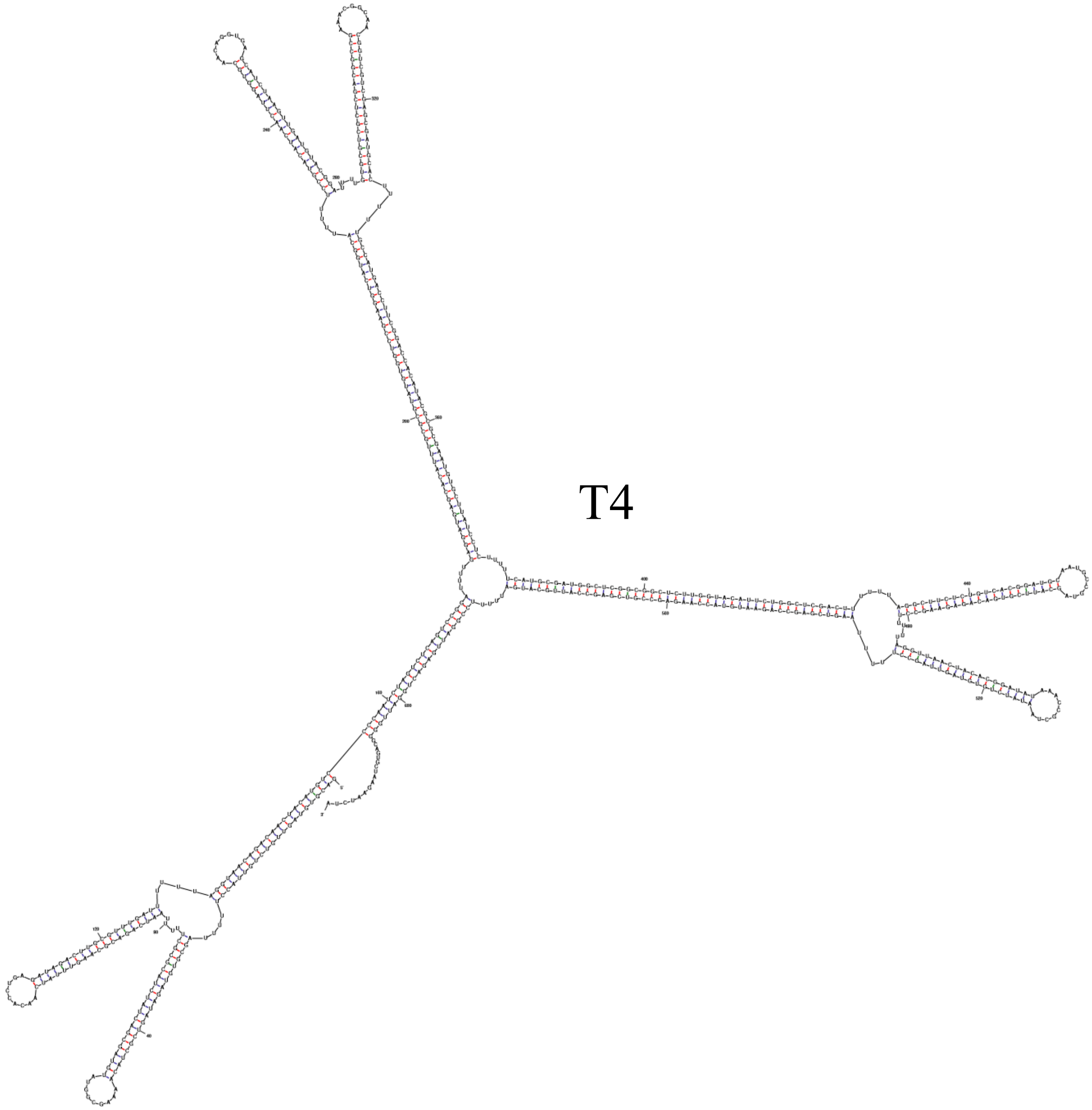

T4*

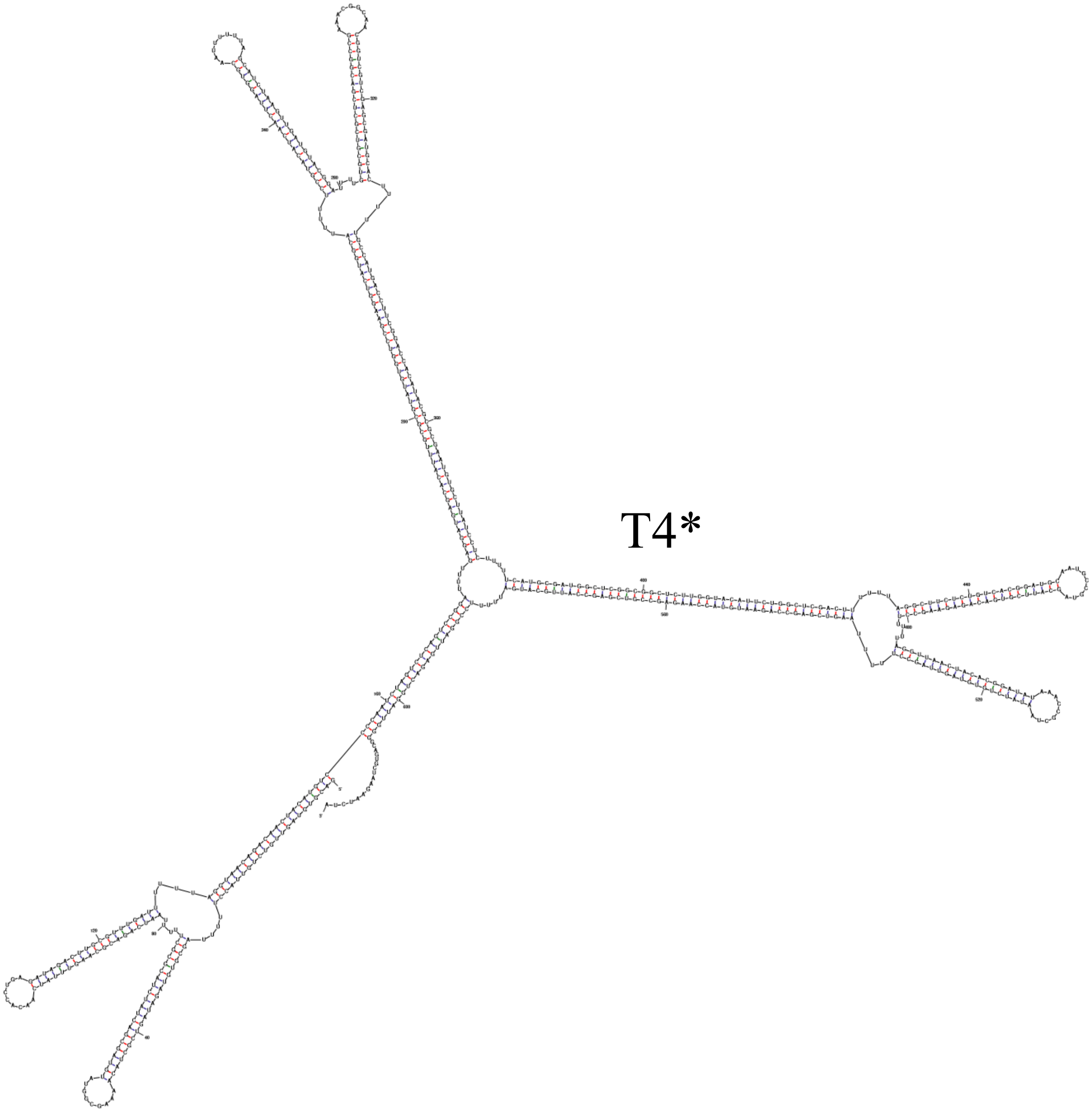

T3

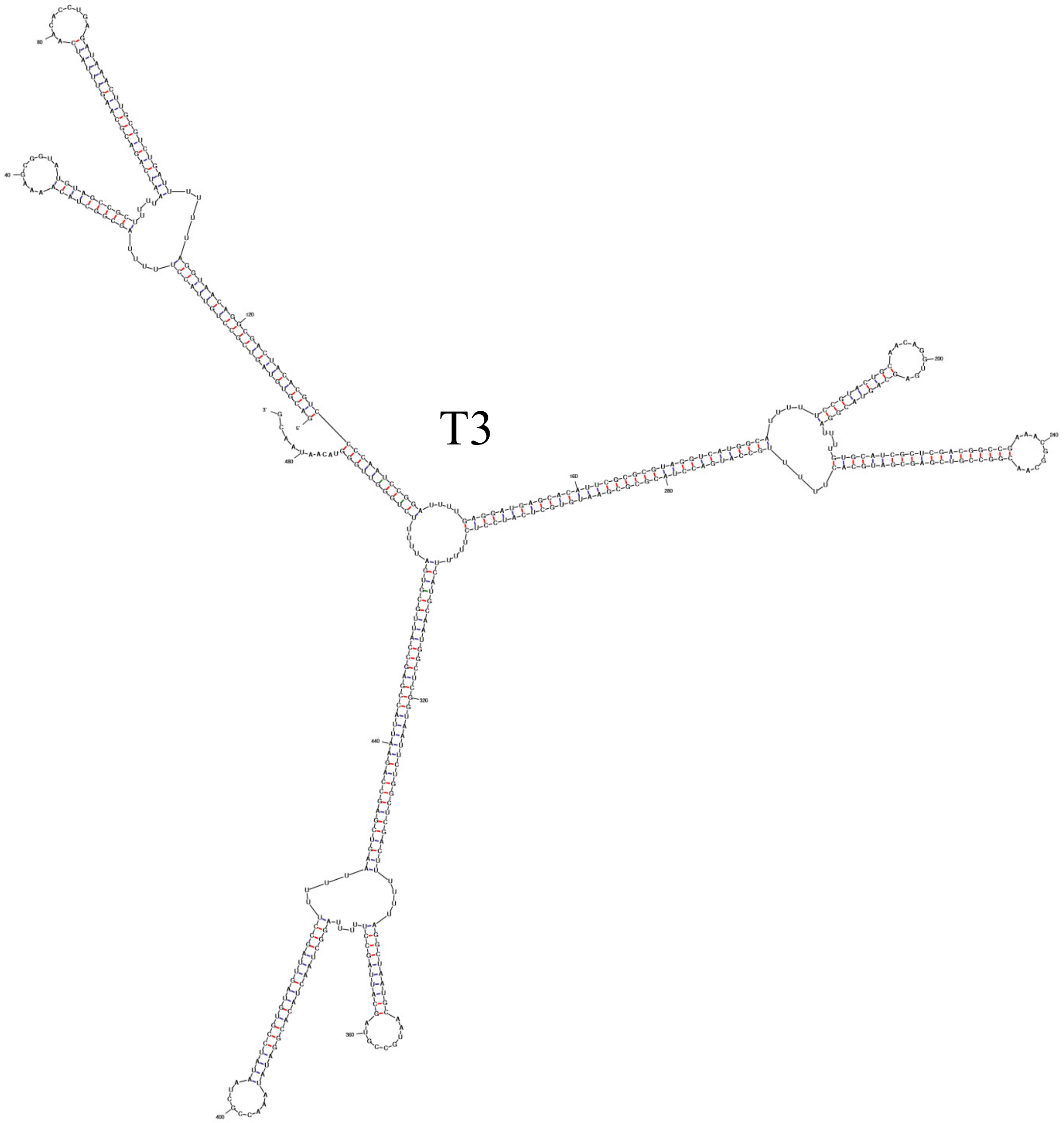

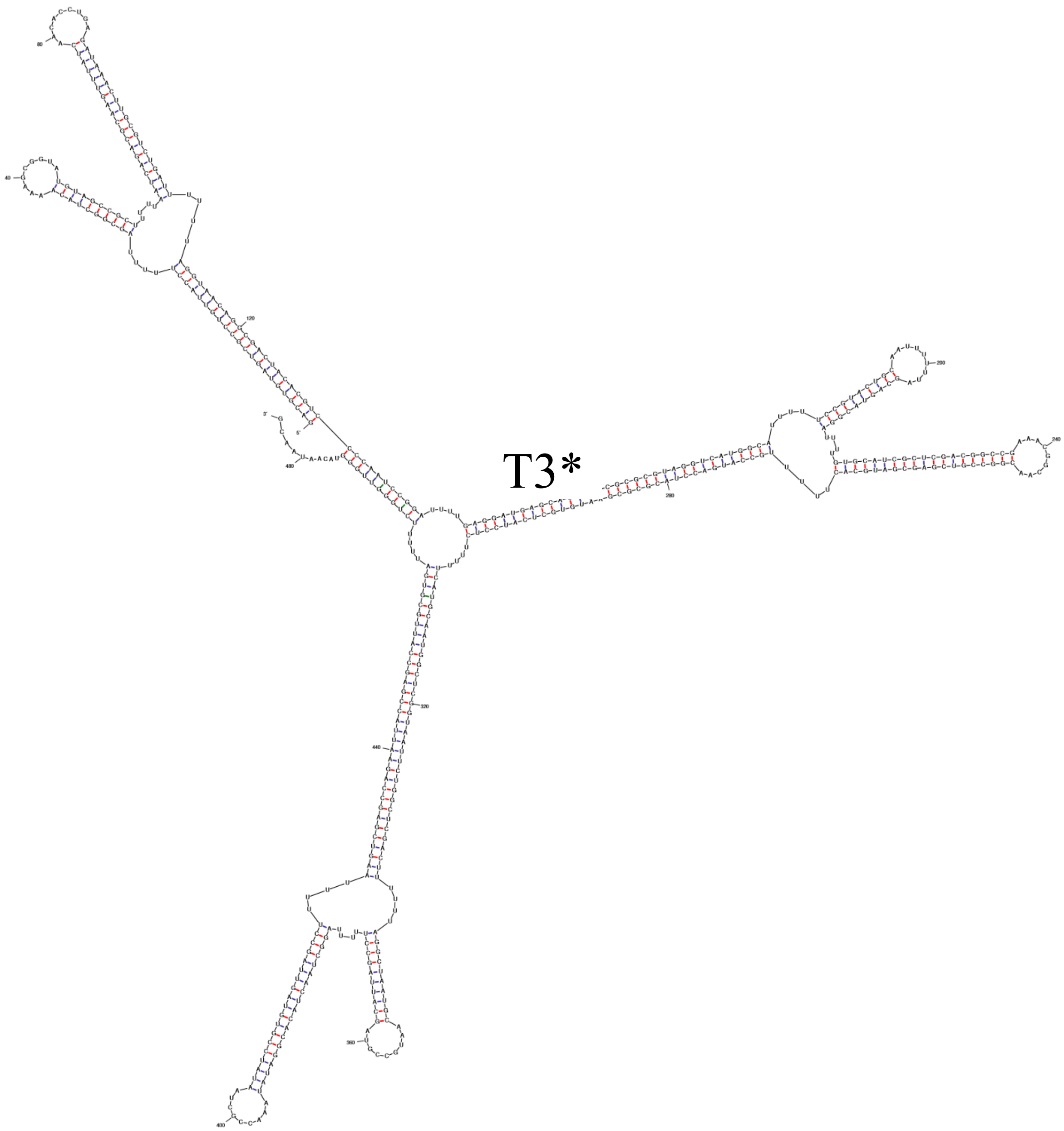

T3*

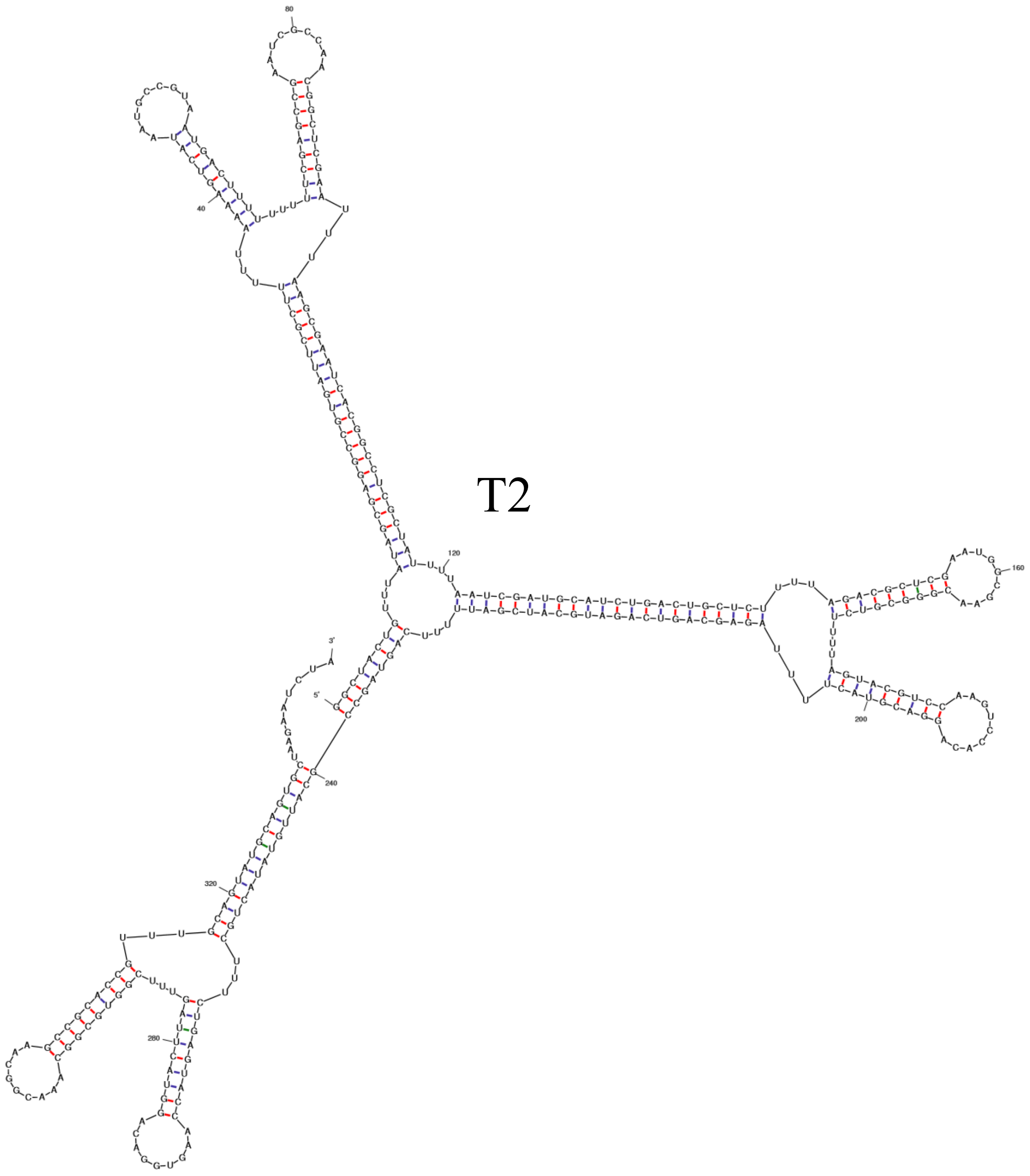

T2

T2*

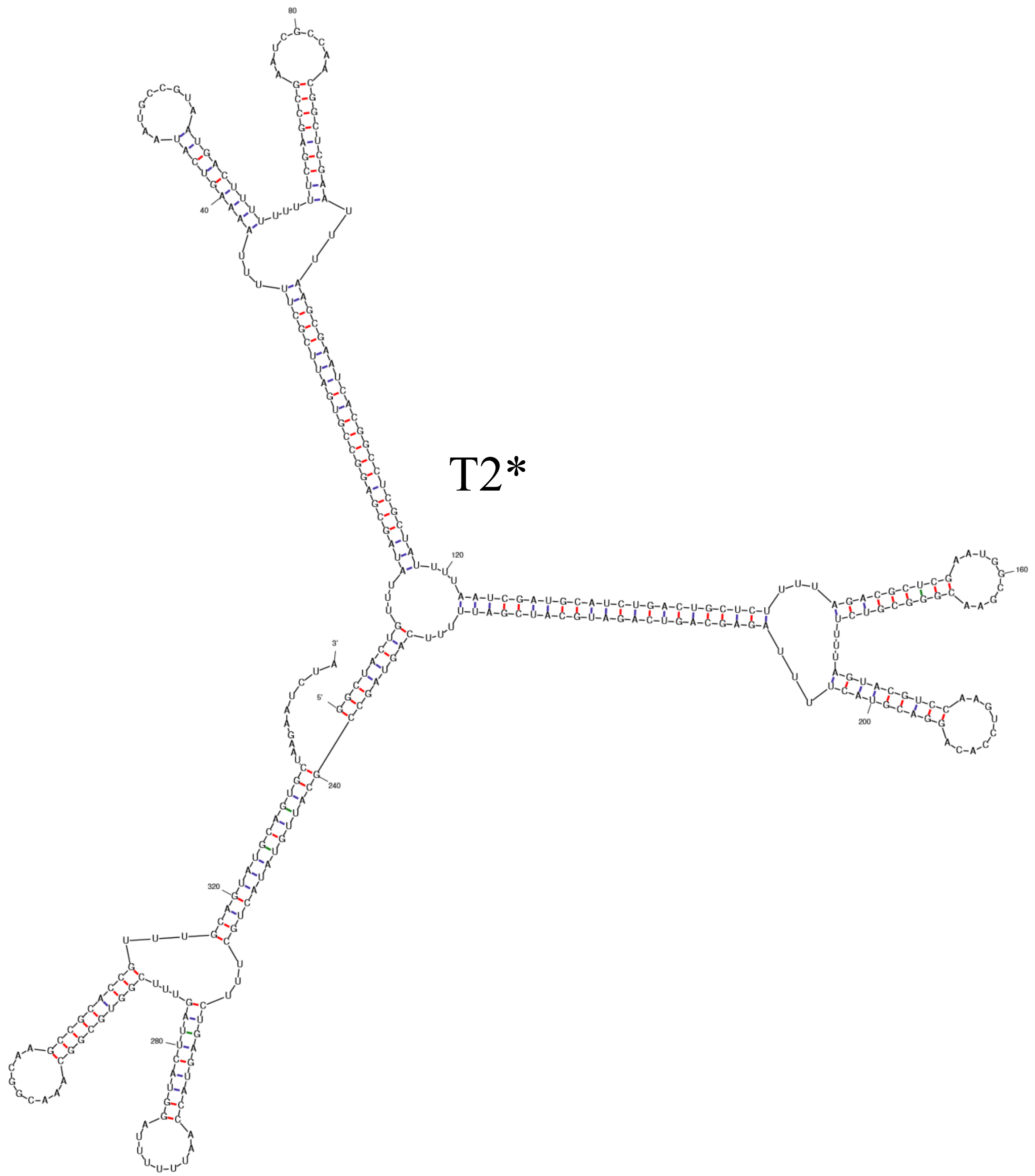

Supplementary Figure 6. RNA tetrahedron: Electrophoretic analysis. For each size, the control molecule (T*) migrates slower than the corresponding tetrahedron molecule (T), suggesting that T* is less compact than T as designed. For each molecule, there is no observable difference in mobility between the quenched and annealed samples, indicating that the RNA folding is fast (faster than the quenching process). The folding efficiency is almost 100%. No bands in T samples show mobilities corresponding to the unfolded T* molecules. All RNA strands used here are PAGE-purified.

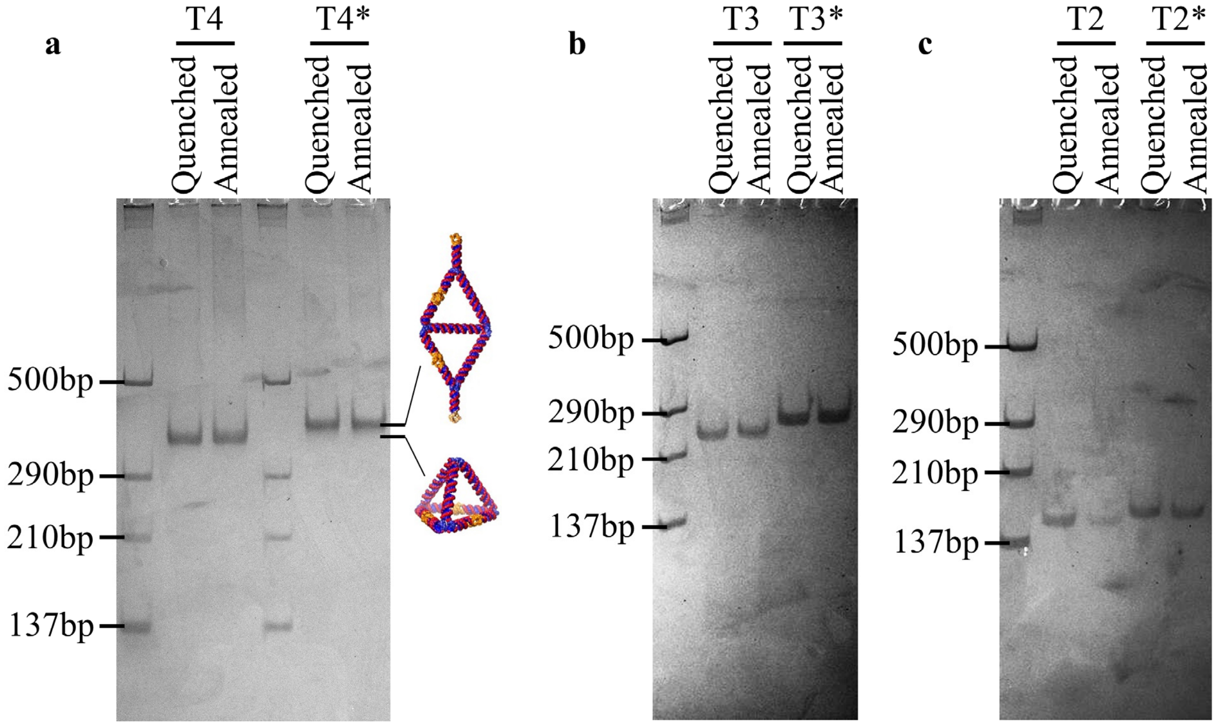

Supplementary Figure 7. Additional AFM images of the RNA tetrahedron. (a) T4 imaged in TAE/Mg^{2+} buffer. (b) T4 and T4* imaged in isopropanol. (c) T2 and T2* imaged in TAE/Mg^{2+} buffer. (d) T3 and T3* imaged in TAE/Mg^{2+} buffer. (Scale Bar: 50 nm)

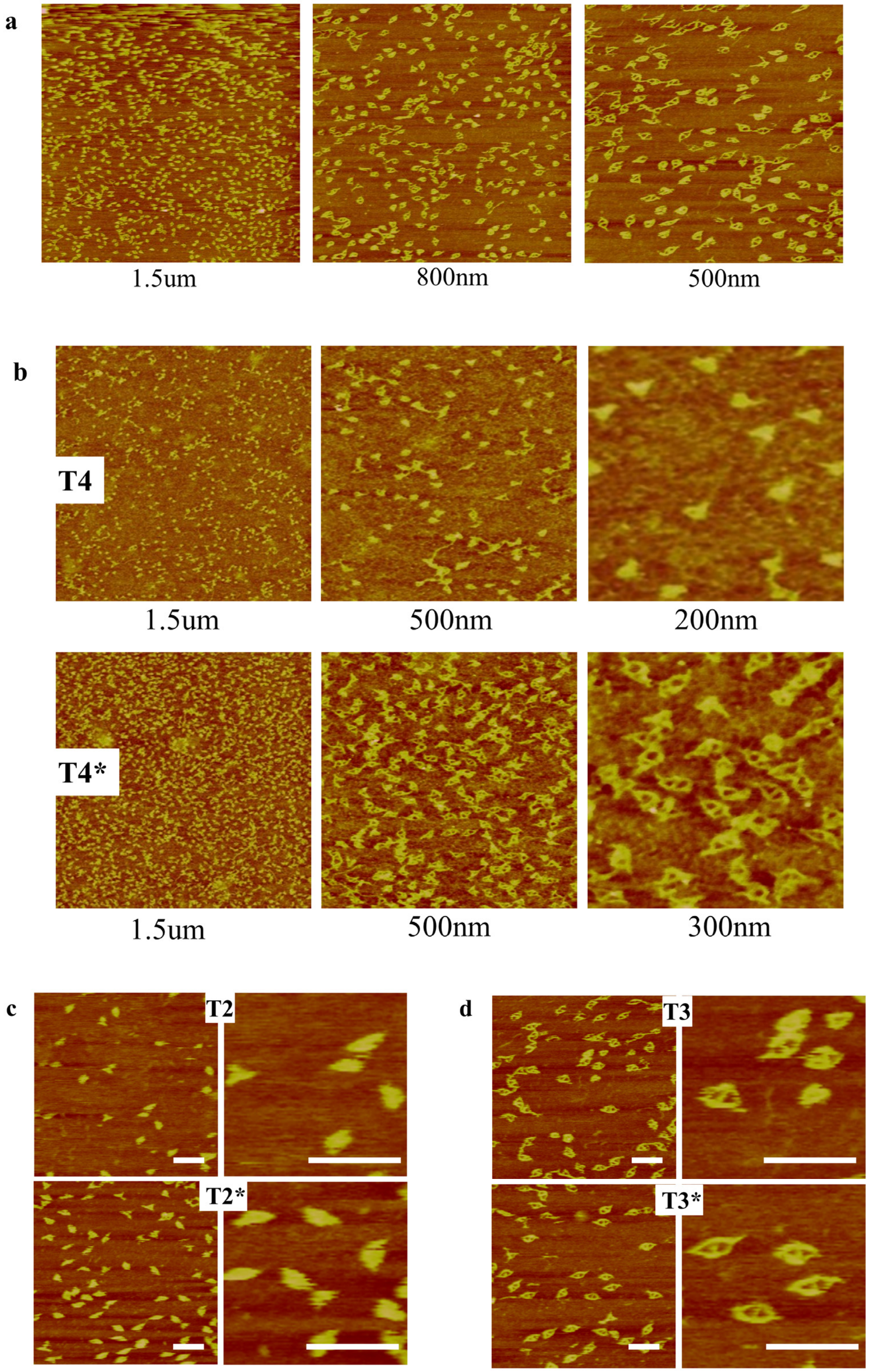

Supplementary Figure 8. CryoEM study of 4-turn RNA tetrahedron (T4). (a) Two raw cryoEM images. (b) Reconstructed initial models from raw particles imposed with different symmetries. (c) Pair-wise comparison of class averages of raw particles (left) with projections of the reconstructed model (right).

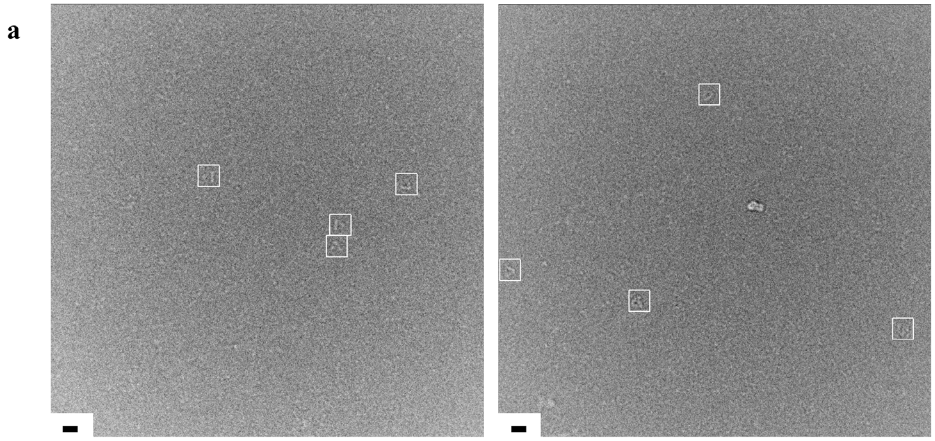

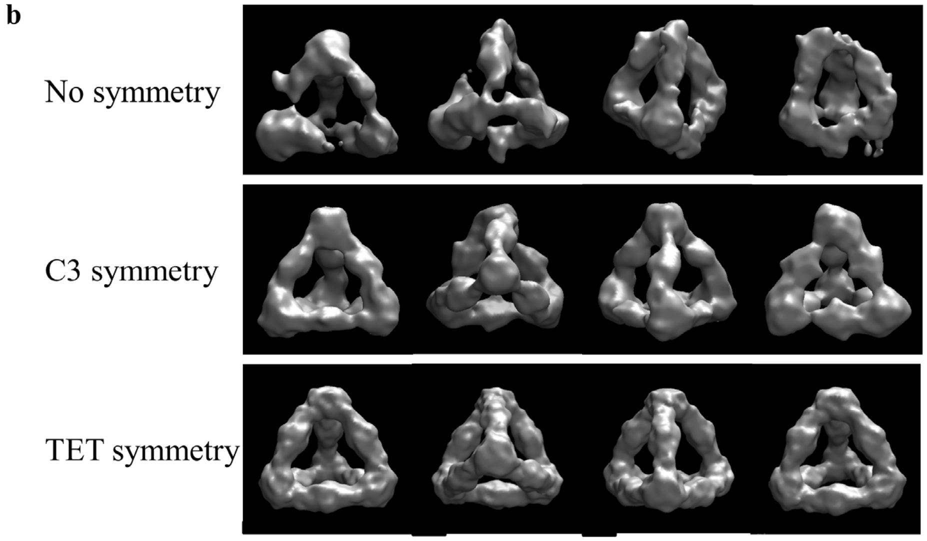

c

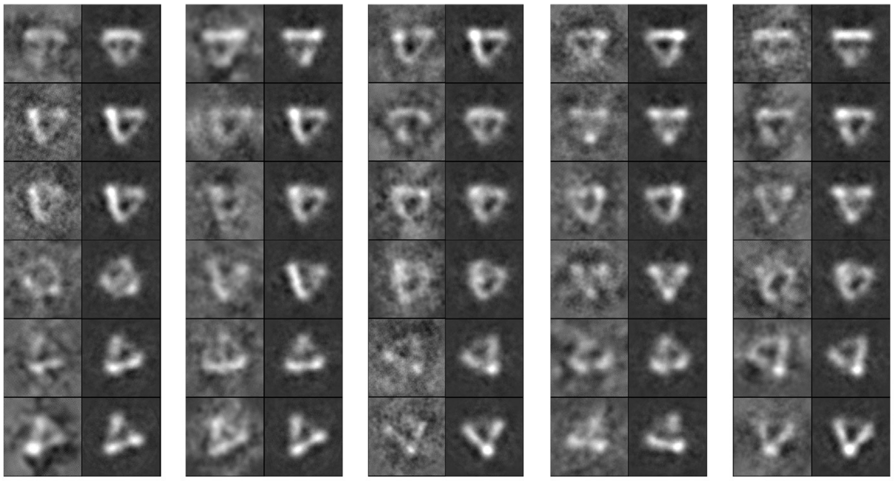

Supplementary Figure 9. Cloning and *in vivo* expression of RNA nanostructure **S2**. (a) A construct of the recombinant expression vector. XbaI and StyI restriction enzymes are the cleavage sites chosen to link the insert and vector. The multiple cloning sites (MCS) between two enzyme sites are deleted. (b) Denaturing PAGE shows the length of expressed RNA (Length of the *in vivo* produced double-square **S2** is 398nt). (c) Native PAGE analysis of the assembly of expressed crude RNA after native extraction from cells. All samples were not annealed except indicating. (B: bacterial RNA, B+P: bacteria with recombinant plasmid without expression induction, B+P+IPTG: with IPTG induction). (d) AFM image of the in vivo expressed **S2**. The cell was lysed by sonication without phenol. Thus, this experiment further confirmed that RNA nanostructure **S2** spontaneously folds in vivo. (Scale Bar: 100 nm)

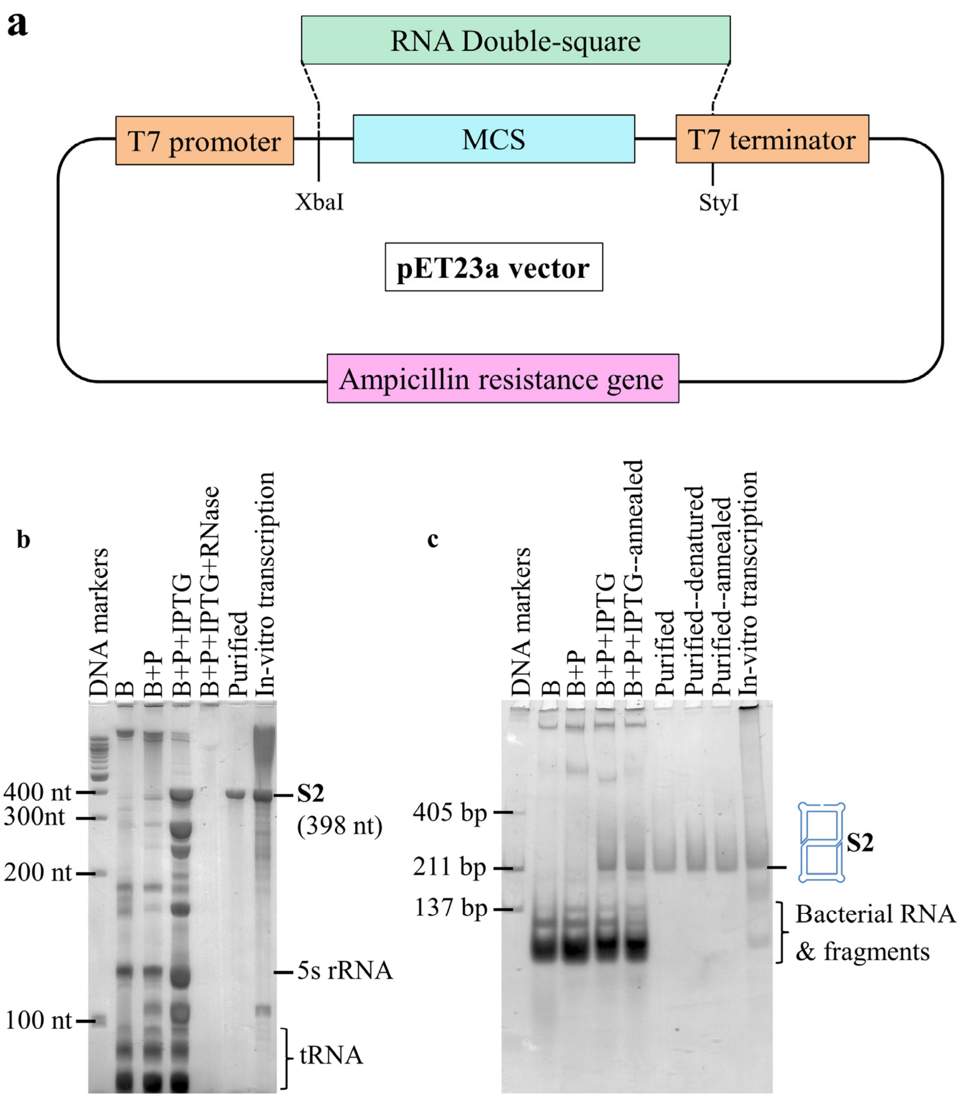

B: Bacteria; P: Recombinant Plasmid

d

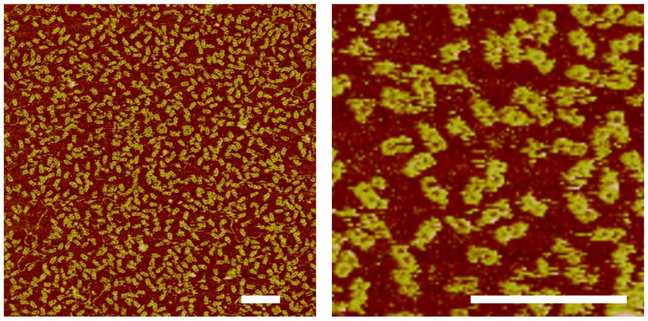

Supplementary Figure 10. Cloning and *in vivo* expression of RNA tetra-square **S4**. (a) A construct of the recombinant expression vector. XbaI and StyI restriction enzymes are the cleavage sites chosen to link the insert and vector. The multiple cloning sites (MCS) between two enzyme sites are deleted. (b) Denaturing PAGE shows the expression of the target **S4** RNA (B+P: bacteria with recombinant plasmid without expression induction, B+P+IPTG: with IPTG induction). (c) AFM image and its close-up views of the in vivo expressed **S4**. (Scale Bar: 20 nm)

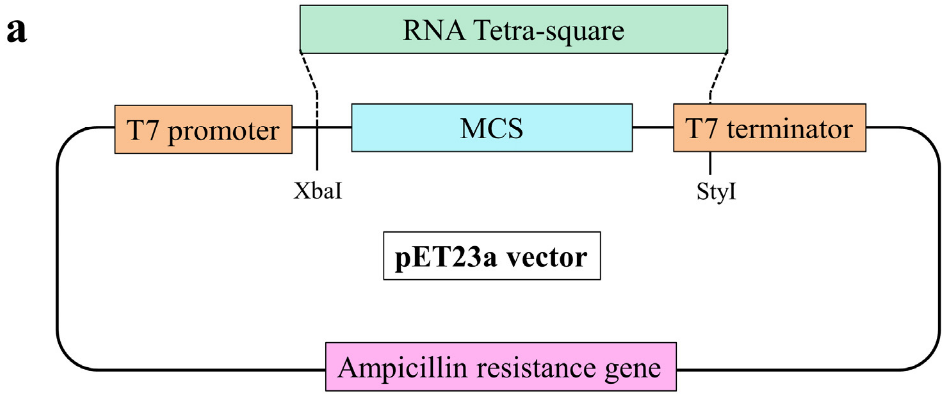

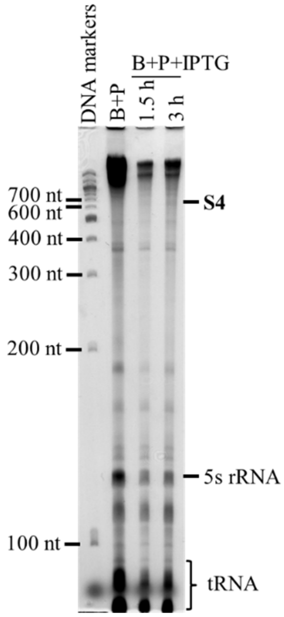

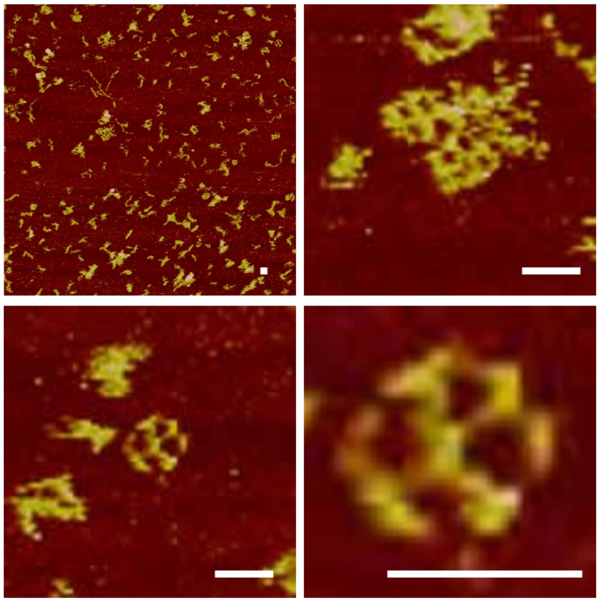

Supplement: Supplementary file 1 — Supplementary Information [file 41467_2018_4652_MOESM1_ESM.pdf]
